# Development of digital measures for nighttime scratch and sleep using wrist-worn wearable devices

**DOI:** 10.1038/s41746-021-00402-x

**Published:** 2021-03-03

**Authors:** Nikhil Mahadevan, Yiorgos Christakis, Junrui Di, Jonathan Bruno, Yao Zhang, E. Ray Dorsey, Wilfred R. Pigeon, Lisa A. Beck, Kevin Thomas, Yaqi Liu, Madisen Wicker, Chris Brooks, Nina Shaafi Kabiri, Jaspreet Bhangu, Carrie Northcott, Shyamal Patel

**Affiliations:** 1 Pfizer, Inc., Cambridge, MA USA; 2 University of Rochester Medical Center, Rochester, NY USA; 3 Department of Veterans Affairs, Canandaigua, NY USA; 4 Boston University School of Medicine, Boston, MA USA

**Keywords:** Atopic dermatitis, Skin manifestations, Drug development, Quality of life

## Abstract

Patients with atopic dermatitis experience increased nocturnal pruritus which leads to scratching and sleep disturbances that significantly contribute to poor quality of life. Objective measurements of nighttime scratching and sleep quantity can help assess the efficacy of an intervention. Wearable sensors can provide novel, objective measures of nighttime scratching and sleep; however, many current approaches were not designed for passive, unsupervised monitoring during daily life. In this work, we present the development and analytical validation of a method that sequentially processes epochs of sample-level accelerometer data from a wrist-worn device to provide continuous digital measures of nighttime scratching and sleep quantity. This approach uses heuristic and machine learning algorithms in a hierarchical paradigm by first determining when the patient intends to sleep, then detecting sleep–wake states along with scratching episodes, and lastly deriving objective measures of both sleep and scratch. Leveraging reference data collected in a sleep laboratory (NCT ID: NCT03490877), results show that sensor-derived measures of total sleep opportunity (TSO; time when patient intends to sleep) and total sleep time (TST) correlate well with reference polysomnography data (TSO: *r* = 0.72, *p* < 0.001; TST: *r* = 0.76, *p* < 0.001; *N* = 32). Log transformed sensor derived measures of total scratching duration achieve strong agreement with reference annotated video recordings (*r* = 0.82, *p* < 0.001; *N* = 25). These results support the use of wearable sensors for objective, continuous measurement of nighttime scratching and sleep during daily life.

## Introduction

Pruritus (itch) is a primary symptom seen in numerous chronic eczematous conditions, especially prevalent in patients with atopic dermatitis (AD)^1^. A common reaction to the pruritus sensation is to scratch the affected area^2,3^, which results in additional inflammation/lesion formation thus exacerbating the pruritus and perpetuating the itch–scratch cycle^4^. Furthermore, pruritus often occurs during the evening and at night and disrupts patients’ sleep^5^. The itch–scratch cycle compounded with sleep disturbances reduces the quality of life of patients as well as caregivers^6,7^.

Traditional assessments of pruritus and sleep are primarily based on clinical outcome assessments (COAs) and patient reported outcome assessments (PROs). COAs are aimed at assessing total body surface area (BSA) of the lesion^8^ as well as lesion severity (redness, induration, excoriations, etc.)^9^ but these are physician-derived measurements and provide limited insight into the fluctuations of symptoms experienced outside the clinic. In contrast, while PROs provide insight into the perceived condition from the patient’s perspective, they are subjective and can be affected by mood or suggestion, lack compliance, and are qualitative in nature^10^. Therefore, there is a need for more objective measures that accurately reflect the impact of AD on a patient’s daily life. These types of measurements not only have the potential to provide more reliable indicators of intervention efficacy, but may also help improve management of the disease.

Advances in wearable sensor technology have already led to more objective measures of health, both within and outside of healthcare settings. Measurement of sleep/wake cycles using wrist-worn accelerometers has continued to evolve since their introduction^11–13^. With improved algorithms, the hope is that actigraphy would provide a more practical and valid option to longitudinally monitor sleep compared to polysomnography (PSG) (the gold standard for sleep assessment)^14^. While PSG provides rich information beyond distinguishing between sleep and wake states (e.g. identifying sleep stages, gross body movements, and respiration patterns), the technically demanding, in-clinic, overnight requirements and cost make it a poor choice for long-term monitoring in situations when these additional parameters are not needed. By leveraging wrist-worn accelerometers, high-resolution measurements can be collected for weeks to months at a time with minimal disturbance to a patient’s daily life. Although methods that rely on accelerometer data are unable to reliably detect sleep stages, they have been used to effectively detect long-term changes in circadian rhythms and sleep quantity^12–14^.

More recently, there have been efforts to leverage data captured using wrist-worn accelerometers in combination with machine learning (ML) techniques to measure nighttime scratching^15–18^. Feuerstein et al.^16^ utilized four signal features derived from accelerometer data with a k-means clustering technique to segment simulated scratching movements (scratching performed on command in a clinic setting) from walking and restless movements during sleep. Petersen et al.^17^ built on this approach by leveraging the same four signal features with logistic regression to also classify simulated scratching movements from walking and restless movements during sleep. However, while both methods achieved high sensitivity in predicting scratch movements (0.90 and 0.96, respectively), because both methods rely on simulated scratching movements and were not designed to support unsupervised monitoring, performance under free-living conditions may suffer. To address some of these limitations, Moreau et al.^18^ trained Recurrent Neural Networks (RNNs) using annotated scratch events during an overnight clinic visit to classify nighttime scratch directly from sample-level accelerometer data. While this approach improves upon previous works by enabling continuous measurement and leveraging data from non-simulated scratch events, it does not segment data into patient’s sleep periods, which can increase the likelihood of false positives during free-living conditions. In addition, while RNNs have proven to be successful with sequence learning tasks^19^, the resulting models often lack interpretability, which is highly valued in regulated environments. Most recently, Ikoma et al.^20^ implemented a heuristic scratch detection approach into a mobile application for use on an Apple Watch (Apple Inc., Cupertino, CA, USA). Since the Apple Watch is a widely used consumer device, this effort represents a considerable step forward toward enabling scalable objective monitoring of patients at home, although the small sample size (*N* = 5) used for algorithm validation as well as the lack of automatic sleep detection are important limitations. While these efforts have certainly advanced the field, there is a need for validated methods that combine both sleep and nighttime scratch detection and allow for deployment under free-living conditions.

Herein, we present a method for continuous, objective assessment of nighttime scratch and sleep based on accelerometer data captured using a wrist-worn wearable device. The proposed method follows a hierarchical paradigm by first determining when the participant intends to sleep, then detecting sleep–wake states and presence of scratch movements, and finally deriving objective measures for both. A pragmatic approach was taken during development of each module in this method, using ML for more complex tasks and heuristic, rule-based algorithms for simpler tasks. Leveraging reference data collected in a sleep laboratory with thermal video scoring (NCT ID: NCT03490877), we examine the performance of each module individually as well as the performance of the proposed method as a whole for continuously deriving endpoints of sleep and nighttime scratch.

## Results

### Participants used for analysis

Of the 45 total AD patients recruited, 12 participants were excluded for algorithm development due to missing accelerometer data, reference thermal video malfunctions, or issues with time alignment between sensor data and reference thermal video, leaving 33 participants (age: 31.1 ± 15.8 [12–63]; sex: 10 (30.3%) male) available for analysis. A table of the relevant data exclusions can be found in Supplementary Table 1. Detailed characteristics of participants used in this analysis are listed in Table 1. Of the available 33 participants, 31 participants had accelerometer data from both wrists, 1 had accelerometer data from the left wrist, and 1 participant did not wear accelerometers on either wrist during the second in-clinic night. A detailed description of the experimental protocol and algorithm development steps for each module in this method can be seen in the “Methods” section.

**Table 1:** Characteristics of participants used for analysis.

Characteristic	Atopic dermatitis patients (*N* = 33)
M/F (*n*)	10/23
Age (years)	31.1 ± 15.8
Race	1 Asian 17 African American 15 White
Body surface area (%)	21.52 ± 22.654
Investigator’s Static Global Assessment	9 Mild 20 Moderate 4 Severe 2.85 ± 0.62
Patient Global Impression of Severity	4.52 ± 1.06
Peak Pruritus Numerical Rating Scale	6.00 ± 2.36
Severity of Pruritus Scale	5 Mild 14 Moderate 14 Severe 2.27 ± 0.72

### Sleep detection performance

As seen in Table 2, we observed almost identical sleep-state detection performance when compared to PSG at the epoch level (30 s) between the left and right wrists. Specifically, the sensitivity and F1 scores for both wrists were consistently high (0.95 and 0.9 for left and right, respectively). There was low observed specificity (0.44 for both left and right) for wake periods.

**Table 2:** Summary statistics on epoch level classification of sleep (30 s) and scratch (3 s).

	Sleep detection (left wrist)	Sleep detection (right wrist)	Scratch detection
	Visit 2 only	Visit 2 only	Visit 1 and visit 2
*n*	32	31	33
Accuracy (mean (SD))	0.85 (0.09)	0.85 (0.10)	0.73 (0.09)
Sensitivity (mean (SD))	0.95 (0.08)	0.95 (0.0 7)	0.61 (0.15)
Specificity (mean (SD))	0.44 (0.24)	0.44 (0.23)	0.80 (0.10)
Positive predictive value (mean (SD))	0.87 (0.11)	0.87 (0.13)	0.73 (0.17)
Negative predictive value (mean (SD))	0.66 (0.24)	0.69 (0.21)	0.68 (0.17)
F1 score (mean (SD))	0.90 (0.07)	0.90 (0.09)	0.66 (0.15)

Epoch level sleep classification performance was assessed using data from the left and right wrist sensors independently. Scratch performance is based on a leave-one-subject-out validation routine, where annotated scratch and restless events from the left and right wrists were pooled together to train a single scratch classifier. Visit 1, visit 2 refer to the two in-lab overnight visits. PSG was available only for visit 2. Mean and standard deviation were computed across participant level performance.

### Scratch detection performance

Across all available in-clinic participant visits, a total of 753.2 min of scratch and restless (non-scratch hand movements; refer to “Methods” section for further details) data (22.8 ± 26.5 min per participant) obtained from both in-clinic visits were used for training the binary ML scratch classifier. This equated to 15,064 distinct 3-s training samples. Epoch level classification performance of the classifier from a leave-one-subject-out validation routine can be seen in Table 2. Per subject classifier performance can be seen in Supplementary Table 2. We observed an improvement in leave-one-subject-out performance of the scratch classifier when using 3 s windows (sensitivity: 0.61 ± 0.15; specificity: 0.8 ± 0.1) compared to 2 s windows (sensitivity: 0.58 ± 0.11, specificity: 0.7 ± 0.08), confirming the decision to use 3 s windows. Using Investigator’s Static Global Assessment (ISGA) scores determined at screening, we observed comparable specificity across AD severity groups (mild: 0.83 (*n* = 9); moderate: 0.80 (*n* = 20); severe: 0.80 (*n* = 4)) and improved sensitivity in patients with severe AD (mild: 0.54 (*n* = 9); moderate: 0.66 (*n* = 20); severe: 0.83 (*n* = 4)). However, statistical tests were not performed because of the small sample size (Supplementary Table 3). In addition, classifier performance by sex showed no clear differences in performance between males and females (Supplementary Table 4). The area under the curve (AUC) of receiver operating characteristics (ROC) was also analyzed to assess the tradeoff between sensitivity and specificity based on the classifier’s prediction threshold. We observed an AUC of 0.81 with the default prediction threshold of 0.5.

### Scratch classifier feature analysis

**Fig. 1:**
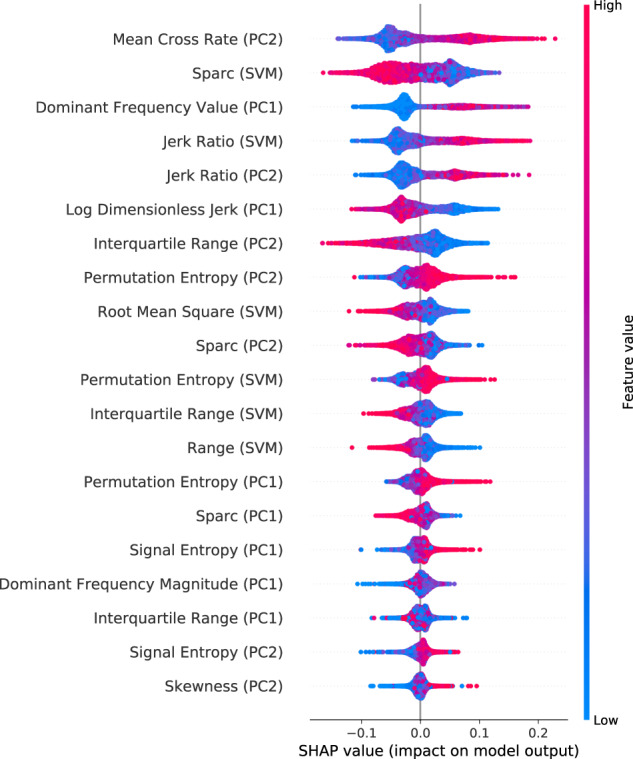
Binary machine learning scratch classifier feature analysis. SHAP^21,22^ analysis was used to assess feature importance and impact on classifier predictions. SVM signal vector magnitude, PC1 first principal component of signal, PC2 second principal component of signal. Features of signal periodicity (e.g. dominant frequency, mean cross rate) and smoothness (e.g. sparc, jerk) are predominant features for distinguishing scratch movements from other restless movements. Further, high values of mean cross rate and dominant frequency increase the likelihood of scratch being predicted, indicating scratching movements involve more rapid and periodic hand movements compared to restless movement. Similarly, high values of jerk and low values of sparc are predictive of scratch, indicating that scratch movements are more irregular and less smooth compared to restless movements.

Figure 1 shows SHapley Additive exPlanation (SHAP)^21,22^ summary values for the top 20 features based on their importance for detecting scratch. We observed that measures of signal periodicity (e.g. dominant frequency, mean cross rate) and smoothness (e.g. sparc, jerk) are the predominant features for scratch detection. Specifically, the mean cross rate of the second principal component signal is the most influential feature. Higher values of this feature (an indication of rapid hand movements) result in higher SHAP values, which indicates a higher probability that the model will predict scratch for the given window. Measures of smoothness (sparc) and dominant frequency are the next two most important features for distinguishing scratch movements from other movements. We observed that higher sparc values (an indication of smoother movements) and lower dominant frequency values (an indication of slower movements) result in a lower probability of scratch prediction by the classifier.

### Evaluation of digital endpoints for sleep and nighttime scratch

We assessed the agreement of aggregate endpoints of sleep (average value of endpoints derived from left and right wrists) and scratch (sum of endpoints derived from left and right wrists) with endpoints derived from PSG and video annotations, respectively. The feasibility of averaging sleep endpoints from the left and right wrists was confirmed by the high agreement observed between left and right-wrist-derived endpoints of total sleep opportunity (TSO), total sleep time (TST), and percent time asleep (PTA) (Pearson correlation coefficient = 0.83, 0.93, 0.94, respectively; all *p*-values < 0.001, see details in Supplementary Fig. 1). Table 3 highlights the Pearson correlation coefficient, statistical significance, and Bland-Altman mean bias and limits of agreement for each endpoint. Thirty-two participants were used for sleep endpoint comparisons (one participant did not wear devices on night 2 and therefore was excluded). In this analysis, TSO derived from PSG was defined as lights-off to lights-on. Significant linear correlations were observed for estimated TSO and TST compared to PSG (*p* < 0.0001). On average, we observed that the sleep module underestimates TSO by 29.7 min and overestimates TST by 24.2 min. Compared with TSO and TST, the linear trend for predicted PTA with PSG is significant but not as strong (*r* = 0.41, *p* = 0.019). Results for exploratory sleep endpoints, specifically number of wake bouts (NWB), wake after sleep onset (WASO), and sleep onset latency (SOL), derived from the binary sleep/wake predictions can be seen in Supplementary Fig. 2. We observed moderate correlations for NWB and WASO. Similar results for single-wrist sleep prediction validation can be seen in Supplementary Fig. 3.

**Table 3:** Agreement of predicted digital summary endpoints with reference system (PSG for sleep, video annotations for scratch) derived endpoints.

Digital measure	Sample size (*n*)	Pearson correlation coefficient	Mean bias and limits of agreement
Total sleep opportunity	32	0.72***	29.66 (−88.63, 147.94)
Total sleep time	32	0.76***	−24.19 (−148.14, 99.75)
Percent time asleep	32	0.41*	−10.17 (−38.89, 18.55)
Total scratch counts (log transformed)	25	0.63***	0.13 (−1.16, 1.41)
Total scratch duration (log transformed)	25	0.82***	0.71 (−0.22, 1.63)

Pearson correlation coefficients along with statistical significance (***p < 0.001, **p < 0.01, *p < 0.05) as well as Bland-Altman limits of agreement are shown.

To evaluate the performance of aggregate nighttime scratch endpoints, we required accelerometer data from both wrists, complete video recordings for a night, and PSG. We compared predicted total scratch counts and duration during the sleep-module-determined TSO against video-annotation-based total scratch counts and duration during the PSG-determined TSO (during night 2 only; when PSG was utilized). Of the 33 participants, 8 participants were excluded (1 participant did not wear the devices during night 2, 1 participant had no accelerometry data for one wrist; and 6 participants had video malfunctions during night 2), leaving 25 participants for this analysis. We observed moderate correlation for scratch counts (*r* = 0.63, *p* < 0.0001) and strong correlation (*r* = 0.82, *p* < 0.0001) for total scratch duration. Additional validation results by pooling scratch counts and durations from night 1 and 2 together (where video annotation data were segmented using the sleep-module-predicted TSO) can be seen in Supplementary Fig. 4, where consistent high correlations were observed. We also observed strong linear correlation between predicted scratch endpoints and WASO (*r* = 0.9, *p* < 0.001 for scratch events, and *r* = 0.82, *p* < 0.001 for scratch durations) and no significant association between scratch endpoints and TST (*r* = − 0.3 for scratch events, and *r* = −0.31 for scratch durations), see details in Supplementary Fig. 5.

## Discussion

**Fig. 2:**
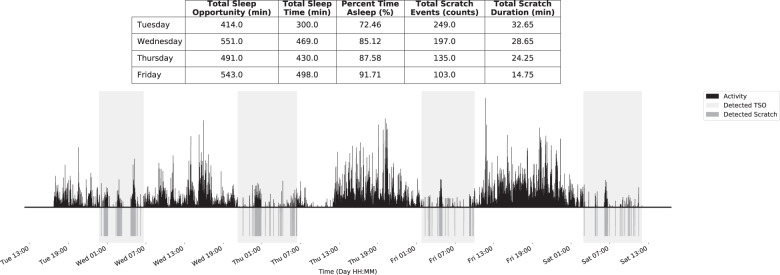
Continuous detection of sleep and nighttime scratch for a 5-day participant recording. The proposed approach is able to process continuous accelerometer recordings from wearable devices worn on the wrist with no input from the user. The total sleep opportunity (TSO) and subsequent scratch events within the TSO period are dynamically detected and assessed for each night of the recording.

We investigated the use of accelerometer and temperature data captured by wrist-worn wearable devices for monitoring sleep and nighttime scratching in patients with AD. The proposed approach was developed with the goal of continuously capturing objective, non-invasive, high-resolution measurements during a patient’s daily life, and enabling scalable deployment in clinical studies. Digital measures of sleep quantity and nighttime scratch showed high correlation with corresponding reference measures of sleep quantity (obtained from PSG) and scratch (obtained from in-clinic video recordings). An example of the continuous measures produced by the proposed approach can be seen in Fig. 2.

An important aspect of the proposed method is the automatic detection of the TSO window. To effectively determine how a subject’s sleep and nighttime scratch vary on a day-to-day basis, the ability to reliably detect the period during which sleep is the intended behavior is paramount. Every endpoint derived by the system relies upon the TSO window to properly bound analysis to the nighttime period. The TSO was chosen over other options, such as the TST window (measured from first sleep epoch to last sleep epoch), with the objective of capturing any difficulties the participant has in falling asleep. While results show that the detected TSO window correlates well with PSG, the system does underestimate the duration by 29.7 min on average. However, the algorithm performance should be viewed in the context of well-known challenges associated with determining TSO boundaries^23^ and the fact that PSG-determined TSO window is set with reference to lights-off/lights-on times rather than actual participant behaviors. For example, we observed based on video recordings that one participant spent until ~3 AM upright in bed working on the computer before lying down to sleep (see Supplementary Fig. 6). The sleep module was able to accurately capture this, but the PSG-based TSO was recorded for the duration between 10 PM and 6 AM. These results remain consistent with previous findings, which saw anywhere from 25–40 min mean absolute error for sleep window duration^24^. The findings with respect to wake epoch detection may in part be related to the relatively small number of PSG-scored wake epochs and is consistent with prior work. Furthermore, the performance of the sleep module (85%) for sleep–wake detection at the epoch level was comparable to that reported by Cole et al.^11^ (88%).

We trained a binary ML classifier using time and frequency domain features extracted during annotated periods of nighttime scratching and restless movements within the TSO window to predict episodes of scratch. SHAP analysis revealed that, of the top five influential features, two are a measure of signal periodicity while the remaining three are a measure of smoothness of movement. Because high values for mean cross rate and dominant frequency increase the likelihood of scratch being predicted, we can infer that scratching movements involve more rapid and periodic hand movements compared to restless movement. Further, high values of jerk-ratio and low values of sparc are predictive of scratch, which suggests that scratching movements are more irregular and less smooth compared to restless movements.

Our approach incorporates hierarchical layers of context detection, simplifying the binary scratch classification task to just determining scratch movements from other restless motion during the night. We observed strong agreement of aggregate endpoints of scratch duration with reference video annotations (*r* = 0.82, *p* < 0.0001) and comparable epoch level scratch detection performance to the approach outlined in Moreau et al.^18^ (total sensitivity: 0.67 vs. 0.66, total F1 score: 0.74 vs. 0.68; see Supplementary Table 2). However, the proposed approach for scratch detection has an added advantage of being more interpretable compared to the RNN/long-short-term-memory neural network approach outlined in Moreau et al.^18^. For example, we analyzed the feature space of participants where the classifier reported low sensitivity and discovered that misclassified scratch windows had slower (lower mean cross rate values) and smoother (lower jerk-ratio and higher sparc values) movements, indicating that the classifier has difficulty in detecting lower intensity movements. These observations align with previous work^18^, and suggest that there may be a ceiling effect on the performance of wrist-based accelerometry to detect scratch. Further research incorporating different sensing modalities (e.g. electromyography (EMG) for measuring activation of muscles, acoustic surveillance, vibration transducers^25^) and locations (e.g. ring sensor for detecting fine finger movements) may be warranted.

While the proposed method achieves good accuracy for detection of scratch events, further work is needed to evaluate the impact of scratching severity (i.e. frequency and intensity of scratching episodes) on the disease state. As discussed above, while the proposed method is more likely to miss low intensity scratching episodes, it is unclear if these episodes contribute significantly to lesion size and the overall patient experience with the disease. Furthermore, when evaluating efficacy of therapeutic interventions in clinical studies, changes in the endpoint values over time rather than their absolute value at a particular moment are more important. Therefore, a consistent bias associated with missing low-intensity scratching episodes may not have a major impact on the reliability of the endpoints for clinical decision making.

Generally, as AD severity increases, the prevalence of sleep disturbances also increase^26,27^, which may be attributed at least partly to increased scratching frequency and intensity. Our results provide some evidence to confirm this hypothesis, as we observed a strong correlation between scratch endpoints and WASO. In contrast, we observed a weak correlation between scratch endpoints and TST. These results indicate that increased nighttime scratching does not necessarily contribute to shorter sleep duration but may result in a more disturbed sleep. Further exploration is needed to better understand this effect.

Prior work suggests that not only scratching, but restless movements (ex. rubbing affected areas on sheets^28^) may contribute to disease progression and AD patients generally exhibit increased restless periods during the night compared to normative populations^28^. Further investigation is needed to better understand how restless movements can affect disease severity, and whether measuring only scratching episodes provides enough information about disease progression to be clinically meaningful. In addition, the increased likelihood of restless behavior in AD populations may also affect the performance of the proposed method by contributing to higher rates of false positives, which may compromise the reliability of digital endpoints of scratch.

Moreover, prior work has also shown a lack of correlation between objective measures of scratch and subjective measures of disease state and itch. Benjamin et al. report minimal agreement between objective measures of scratch and parental assessments of itch^28^. Similar results have been reported for patient reported measures of itch (Visual Analogue Scale)^10^, six area, six sign atopic dermatitis (SASSAD) severity score, patient oriented eczema measure (POEM), or the dermatitis family impact (DFI) score^29^. We interpret these results as evidence of a disconnect between objective, accelerometry-based measures of scratch and subjective measures of itch. While the terms itch and scratch are often used interchangeably, they are different phenomena. Itch is a feeling with complex neurological underpinnings that may result in the action of scratching, and this distinction between perception and behavior is one that merits further investigation. Additional studies applying this method longitudinally are needed to better understand the relationship of these objective endpoints to changes in disease state in patients with AD.

Scalability was an important factor that we took into account for the design of the proposed method. In order to maximize generalizability, we imposed a constraint that the solution be device agnostic. This meant building algorithms that operate solely on sample-level data directly, instead of proprietary data streams that numerous wearable devices today output. Additionally, we minimized reliance on device orientation (as sensor components might be oriented differently from device to device) by performing transformations and dimensionality reduction on the sample-level data where possible. In order to minimize the number of times patients must remove the device for the purpose of charging the battery, we focused on using accelerometer and temperature data sampled at 20 Hz to maximize battery life and believe these choices will also improve patient compliance. Along with that, the proposed method allows for the possibility of deploying a single-device solution by processing each wrist independently. As a result of these design choices, the proposed solution offers the flexibility to use devices and deployment strategies that best fit the use-case while minimizing patient burden.

Although utilizing hierarchical layers of context detection simplifies the downstream classification tasks, there is a risk of error propagation. While the performance of the proposed solution was promising based on data collected in the clinic, application on data collected at home for longer durations (weeks or months) is necessary to investigate generalizability. Along with that, accurate on-body detection while monitoring under daily-life conditions is critical to ensure reliability of the endpoints. The proposed solution relies on near-body temperature to detect and exclude non-wear periods from analysis, which limits its application to devices that either have that sensing capability or provide another approach (e.g. capacitive touch sensing) for robust detection of non-wear periods. A future direction could also focus on assessing the proposed digital endpoints’ sensitivity for detecting clinically meaningful changes associated with disease progression or therapeutic interventions.

## Methods

### Participants

Forty-five AD patients were recruited as part of a larger research effort (determined by the criteria of Hanifin and Rajka, the ISGA (≥2), and BSA (≥5%) obtained at screening (0–30 days prior to visit 1); aged 31.7 ± 16.01 years [12–63; range]; sex: 16 (35.5%) male). Participants were also required to have active pruritus as determined by PROs: Peak Pruritus Numerical Rating Scale (ppNRS^30^ (Instrument copyrighted by Regeneron and Sanofi-Aventis); ≥3) and Severity of Pruritus Scale (SPS; ≥1) at screening, and permitted to continue concomitant AD treatments during the study. The algorithm development and validation presented in this work is derived from a subset of data collected in the larger research study (12 participants excluded due to missing data required for algorithm development and evaluation).

### Study design and experimental protocol

Participants were monitored for a total of four nights: two nights in a sleep laboratory (in-clinic) and two nights at home. During each in-clinic visit, participants were monitored throughout the night via infrared radar thermal videography (FLIR A35; Flir Systems, Wilsonville, OR, USA) in a sleep lab. Video recordings of each participant were subsequently reviewed by two trained human annotators to identify the presence of scratch and restless movements and a final arbitrator if there were disagreements on the assessments. Further details about the scratch and restless movement criteria used to define and annotate scratch behaviors (developed by Boston University School of Medicine Laboratory for Human Neurobiology) are in Supplementary Note 1. In addition to videography, during the second overnight clinic visit, participants’ sleep was monitored via limited PSG. Following the in-clinic visit, participants were monitored for two nights at home. All procedures in this study had approval from the University of Rochester Medical Center Institutional Review Board. All participants in the study gave written informed consent prior to enrollment. Data from both in-clinic visits were used for scratch algorithm development and data from the second in-clinic visit were used for sleep algorithm development.

### Instrumentation

Participants wore two devices (GeneActiv Original; Activinsights, Kimbolton, UK), one on each wrist, during both in-clinic and at-home visits. These devices have a watch-like form factor (although no watch face) and are designed for continuous, multi-day recordings in both free-living and clinical environments. Sample-level sensor data (triaxial acceleration, near-body temperature, and ambient light) is logged on the device and can be downloaded at the end of the monitoring period. In this study, data from a triaxial accelerometer (sampling rate: 100 Hz, unit: g), ambient light sensor (sampling rate: 100 Hz, unit: Lux), and near-body temperature sensor (sampling rate: 0.334 Hz, unit: Celsius) were collected. Participants were instructed to wear the devices at least 3 h prior to the first overnight clinic visit and leave them on throughout the evaluation period (i.e. not remove during the day). During the second overnight clinic visit, participants underwent PSG, which was used as the ground truth measurement of sleep. PSG recordings consisted of external electrodes (three electroencephalography (EEG) sites (C3, C4, and Occipital), two electrooculography (EOG) sites, two facial EMG sites, reference electrodes, and ground) that were placed on the head and face (one on either side of each eye, one behind each ear, and two on the chin/jawline). PSG recordings did not include measurement of respiration, limb movement, or oximetry, and were scored in 30-s epochs per revised American Academy of Sleep Medicine scoring guidelines^31^. Both in-clinic visits included thermal videography recorded in 72,000 frame batches at 60 Hz (equating to 20 min per video) for the duration of the subject’s overnight visit.

### Analytical approach

**Fig. 3:**
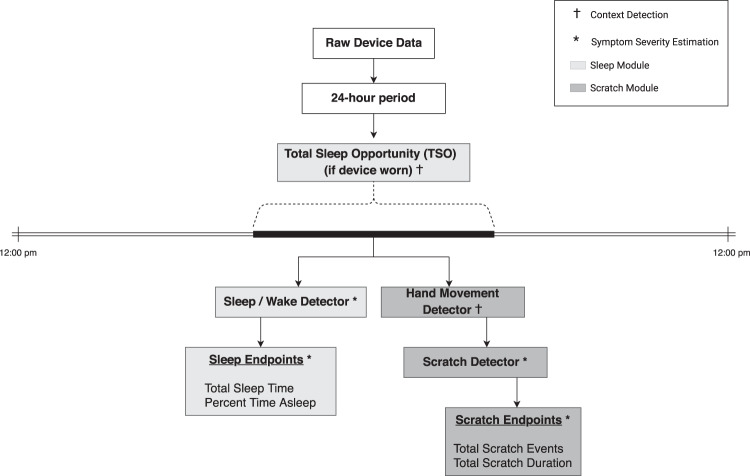
Flowchart highlighting hierarchical approach for detection and assessment of sleep and nighttime scratch using accelerometer data from a wrist-worn device. Raw accelerometer data are first sliced to a 24-h period (12:00 PM–12:00 PM), then segmented to the total sleep opportunity (TSO) window, and finally measures of sleep and scratch are computed during the TSO window.

As illustrated in Fig. 3, the proposed method for assessment of nighttime scratch and sleep follows a hierarchical paradigm by first performing context detection and then estimating symptom severity. Context detection consists of wear detection (on-body vs. off-body), detection of the subject’s TSO window (defined as the largest period in a 24-h window during which sleep is the intended behavior, or more simply the time from when the participant laid down to go to bed to the time when they rise in the morning), and detection of hand movement during the predicted TSO window. Symptom severity estimation consists of detection and assessment of sleep quantity and nighttime scratch. In order to evaluate sleep and scratch independently, we separate the method into two modules: (1) sleep module, consisting of the wear detector, TSO detector, sleep/wake detector, and sleep assessment, and (2) scratch module, consisting of the hand movement detector, scratch detector, and scratch assessment. The development procedure for each module of the method is explained in detail below.

### Sleep module

**Fig. 4:**
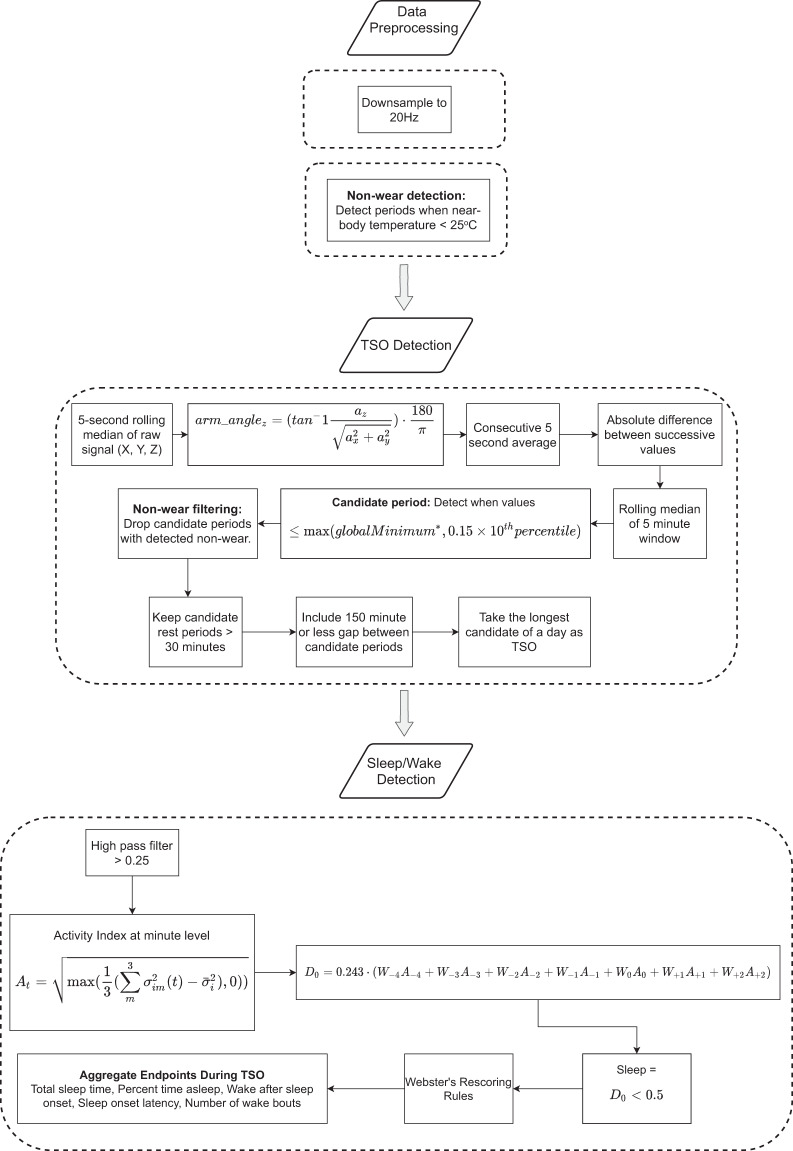
Overview of the sleep module processing pipeline. The pipeline consists of data preprocessing, total sleep opportunity (TSO) and wear detection, sleep/wake classification, and sleep assessment. *Global minimum is set to 0.1 based on the 25th percentile value of all valid (on-body) data (see Supplementary Fig. 7). Block with a dashed outline provides a detailed illustration of steps in the preceding block with a solid outline.

The sleep module incorporates several previously published algorithms^11,24,32^ in a modular framework to provide measures of sleep quantity^33^. An overview of the processing pipeline can be seen in Fig. 4.

Accelerometer data obtained from the wrists were first down sampled from 100 Hz to 20 Hz. Data were then separated into 24-h segments (12:00 PM to 12:00 PM the next day). Any 24-h periods with less than 6 h of recording time were discarded. This was done to exclude data recorded before and after the official visit period, incomplete data, or data from a misconfigured device (each recording was expected to be approximately 48 h). Periods of non-wear were determined by applying a heuristic rule to the near-body temperature data recorded by the GeneActiv Original device. The temperature data were first processed similarly to the sample-level accelerometer data (5-s rolling median, consecutive 5-s average, rolling 5-min median) so that the wear/non-wear periods would be aligned with the candidate TSO periods. Any candidate period with a temperature value less than 25 °C was considered non-wear. The 25 °C threshold was empirically derived from known wear data during sleep (Supplementary Fig. 7).

Candidate TSO periods for each 24-h segment were then determined using a heuristic approach based on change in arm angle calculated using accelerometer data from the wrist^24^. Any candidate TSO period that was classified as non-wear was excluded. Of the remaining candidate TSO periods in a given 24-h segment, the longest one was chosen as the TSO window. Once the TSO window was identified, predictions of sleep and wake were generated for each 1-min epoch using a heuristic approach^11^. The previously published sleep–wake classification algorithm that was implemented relies on a proprietary method to derive activity counts, which were not available for the wearable device used in this study. Therefore, an open-source activity index metric^32^ was used as a proxy for activity counts in our implementation. Webster’s rescoring rules were applied to the binary sleep–wake predictions to improve specificity^11^. Digital measures of sleep were derived for each 24-h segment by processing the sleep–wake predictions during the determined TSO window as seen in Table 4.

**Table 4:** Description of digital measures output from the analytics solution.

Digital measure	Type	Units	Description
Total sleep opportunity (TSO)	Sleep	Minutes	Largest window of time where sleep is the intended behavior.
Total sleep time (TST)	Sleep	Minutes	Total time spent asleep during the total sleep opportunity window.
Percent time asleep (PTA)	Sleep	Percentage	Percentage of the total sleep opportunity window spent in the sleep state.
Total scratch events	Scratch	Counts	Total scratch bouts during the total sleep opportunity window.
Total scratch duration	Scratch	Minutes	Total time scratching during the total sleep opportunity window.

### Scratch module

**Fig. 5:**
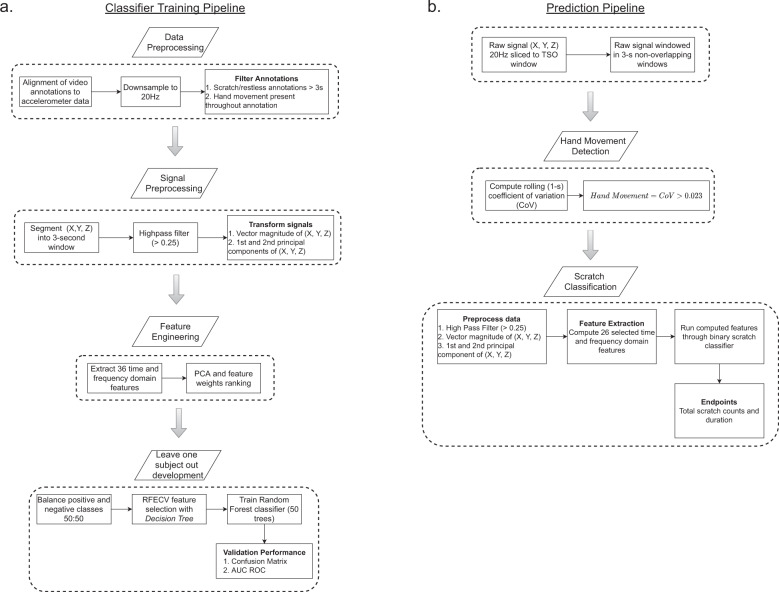
Overview of the prediction and classifier training pipeline for the scratch module. **a** Scratch classifier training pipeline, consisting of data preprocessing, signal preprocessing, feature engineering, and leave-one-subject-out validation. **b** Scratch module prediction pipeline, consisting of data preprocessing, hand movement detection, scratch classification, and scratch assessment.

Predictions of nighttime scratch were generated via a two-tiered approach (Fig. 5b). First, the presence of hand movement was determined, and then those periods of hand movement were classified as either scratch or non-scratch events. Sample-level accelerometer data were segmented into 3-s non-overlapping windows within the selected TSO window for a given 24-h period. After testing multiple window lengths (1, 2, and 3 s), we found that a 3-s window achieved a good tradeoff between temporal resolution and detection performance. This choice is in agreement with prior work on scratch classification^16,17^ and human activity recognition^34^. Each 3-s window was passed through a heuristic hand movement detection algorithm^35^ to determine the presence of hand movement. The primary parameter of the hand movement algorithm (a threshold applied to rolling coefficient of variation) was tuned empirically based on our dataset. After testing several threshold values, we selected the 25th percentile of the distribution of coefficient of variation values (0.023) based on our dataset. Scratch classification was performed on a 3-s window only if hand movement was present for the entirety of the window.

We trained a binary ML classifier to detect the presence of scratch. For training, we used all available 3-s windows across both in-clinic visits. To generate labels for training the classifier, instances of nighttime scratch and restless (non-scratch) movements observed via thermal videos of each in-clinic participant visit were annotated by human raters using criteria outlined in Supplementary Note 1. Annotations were performed by two annotators and reviewed by an arbitrator if there were disagreements with regard to timing or behavior classification. Each annotation included metadata about which hand was moving (right, left, or both), the affected body location, as well as severity of scratching (mild, moderate, severe; see Supplementary Note 1 for definitions). Annotations of 3 s or longer were used for training the binary classifier. If an annotation was greater than 3 s, it was segmented into 3 s windows with 50% overlap prior to training to maximize data availability. To ensure that the ground truth was reliable, all annotations were manually time-aligned with the accelerometer data based on a prescribed clap event (participant instructed to clap in front of camera while wearing accelerometer devices) during each in-clinic visit.

The pipeline for training the binary scratch classifier included steps for preprocessing, feature extraction, feature selection, model training, and model evaluation (Fig. 5a). The preprocessing step generated three processed signals by applying filtering and dimensionality reduction to the sample-level accelerometer data. First, the data were filtered using a first-order Butterworth infinite impulse response (IIR) high-pass filter with a cutoff frequency of 0.25 to remove acceleration due to gravity. Next, to reduce dependence on device orientation, the signal vector magnitude (SVM) $( {\sqrt {x^2 + y^2 + z^2} })$ as well as the first (PC1) and second (PC2) principal components of the filtered signal were computed.

A total of 36 time and frequency domain features were then calculated from the preprocessed signals for each window (Supplementary Table 5). Observations were then randomly sampled to balance the positive and negative classes prior to feature selection. We performed feature selection using recursive feature elimination with cross-validation with a decision tree as the estimator^36^, which resulted in a total of 26 selected features. A random forest classifier with 50 estimators was then trained based on the selected features. Performance of the binary model was assessed using a leave-one-subject-out validation. We evaluated multiple settings for number of estimators in the random forest classifier (25, 50, 75, and 100) and saw no significant improvement in model performance as we increased the number of estimators past 50.

Digital measures of nighttime scratch were derived by processing the binary scratch predictions during the predicted TSO window for each 24-h segment (Table 4). Total scratch counts were computed by taking the sum of contiguous 3-s bouts of predicted scratch detected from both wrists. Total scratch duration was computed by taking the sum of the duration of all predicted scratch bouts from both wrists.

### Statistical methods to measure agreement between sensor-derived measures and reference data

Performance of both the sleep and scratch algorithms were evaluated at the epoch (30 s and 3 s, respectively) and summary endpoint (summary metrics seen in Table 4) levels. With the aim of transitioning to a single device setup in the future, epoch predictions of sleep were derived from both the left and right wrists and were assessed independently against PSG during the second in-clinic visit. A leave-one-subject-out validation was used to assess scratch performance at the epoch level. Annotated scratch and restless movements from the left and right wrists during both in-clinic visits were pooled together to train a single scratch classifier. Conventional classification performance metrics (accuracy, sensitivity, specificity, F1 score, and AUC of the ROC) were used to summarize epoch level performance for both sleep and scratch modules. SHAP^21,22^, a game theoretic approach, was used to analyze the importance of the selected features used in the ML scratch classifier.

Epoch predictions of sleep and scratch (with scratch based on leave-one-subject-out model predictions) are summarized for each participant night to obtain endpoint level predictions. Summary statistics of scratch algorithm performance are calculated for different ISGA severities (taken at screening) and sex. Pearson correlation coefficients (along with their *p*-values), Bland-Altman plots, and limits of agreement were used to assess agreement with reference data on endpoint level metrics throughout. The Bland-Altman limits of agreement describe the 95% confidence interval between the measurements being compared. The agreement between sensor-derived sleep endpoints from the left and right wrists was also assessed. Subsequently, aggregate endpoints of sleep (derived by taking the average between the left and right wrists) were assessed against PSG-derived endpoints of sleep during the second in-clinic visit. Aggregate endpoints of scratch (derived by taking the sum of left and right wrist outputs) during the TSO window predicted by the sleep module were assessed against video annotation derived endpoints of scratch during PSG determined TSO window. Since the distributions of sensor-derived scratch endpoints were right-skewed, log transformation was applied (specifically, log(*x* + 1) to include possible zero values).

### Reporting summary

Further information on research design is available in the Nature Research Reporting Summary linked to this article.

## Supplementary information

Reporting Summary

Supplementary Information

## Data Availability

Upon request, and subject to review, Pfizer will provide the data that support the findings of this study. Subject to certain criteria, conditions, and exceptions, Pfizer may also provide access to the related individual anonymized participant data. See https://www.pfizer.com/science/clinical-trials/trial-data-and-results for more information.

## Code availability

The sleep module outlined in this publication is available on GitHub: https://github.com/elyiorgos/sleeppy.
