# Supplementary material for: Development of digital measures for nighttime scratch and sleep using wrist-worn wearable devices
Source: NPJ Digit Med. 2021 Mar 3;4:42. doi: 10.1038/s41746-021-00402-x (PMC7930047; doi:10.1038/s41746-021-00402-x)
Supplement: Supplementary file 1 — Reporting Summary [file 41746_2021_402_MOESM1_ESM.pdf]

nature research

Corresponding author(s): Nikhil Mahadevan

Last updated by author(s): Nov 24, 2020

Reporting Summary

Nature Research wishes to improve the reproducibility of the work that we publish. This form provides structure for consistency and transparency in reporting. For further information on Nature Research policies, see our Editorial Policies and the Editorial Policy Checklist.

Statistics

For all statistical analyses, confirm that the following items are present in the figure legend, table legend, main text, or Methods section.

n/a	Confirmed	
☐	☒	The exact sample size (*n*) for each experimental group/condition, given as a discrete number and unit of measurement
☒	☐	A statement on whether measurements were taken from distinct samples or whether the same sample was measured repeatedly
☐	☒	The statistical test(s) used AND whether they are one- or two-sided<br>*Only common tests should be described solely by name; describe more complex techniques in the Methods section.*
☐	☒	A description of all covariates tested
☐	☒	A description of any assumptions or corrections, such as tests of normality and adjustment for multiple comparisons
☐	☒	A full description of the statistical parameters including central tendency (e.g. means) or other basic estimates (e.g. regression coefficient) AND variation (e.g. standard deviation) or associated estimates of uncertainty (e.g. confidence intervals)
☐	☒	For null hypothesis testing, the test statistic (e.g. *F*, *t*, *r*) with confidence intervals, effect sizes, degrees of freedom and *P* value noted<br>*Give P values as exact values whenever suitable.*
☒	☐	For Bayesian analysis, information on the choice of priors and Markov chain Monte Carlo settings
☐	☒	For hierarchical and complex designs, identification of the appropriate level for tests and full reporting of outcomes
☐	☒	Estimates of effect sizes (e.g. Cohen's *d*, Pearson's *r*), indicating how they were calculated

Our web collection on statistics for biologists contains articles on many of the points above.

Software and code

Policy information about availability of computer code

Data collection	GENEActiv original devices (https://www.activinsights.com/products/geneactiv/) were used to collect accelerometry data used in our experiments. The FLIR A35 thermal camera (https://www.flir.com/products/a35/) was used to produce overnight video recordings. Data related to patient reported outcomes, clinical assessments, demographics, and timestamps associated with protocol activities were captured using Encapsia by Cmed. (https://www.encapsia.com/)
Data analysis	Python and various open source modules were used to implement the data processing pipeline. The code used for sleep analysis is available here: https://github.com/elyiorgos/sleeppy

For manuscripts utilizing custom algorithms or software that are central to the research but not yet described in published literature, software must be made available to editors and reviewers. We strongly encourage code deposition in a community repository (e.g. GitHub). See the Nature Research guidelines for submitting code & software for further information.

Data

Policy information about availability of data

All manuscripts must include a data availability statement. This statement should provide the following information, where applicable:

- Accession codes, unique identifiers, or web links for publicly available datasets
- A list of figures that have associated raw data
- A description of any restrictions on data availability

The datasets used in this study are not publicly available because they contain protected patient health information.

Field-specific reporting

Please select the one below that is the best fit for your research. If you are not sure, read the appropriate sections before making your selection.

☐ Life sciences ☒ Behavioural & social sciences ☐ Ecological, evolutionary & environmental sciences

For a reference copy of the document with all sections, see nature.com/documents/nr-reporting-summary-flat.pdf

Behavioural & social sciences study design

All studies must disclose on these points even when the disclosure is negative.

Study description	The study was designed as a quantitative, observational experiment in which patients with Atopic Dermatitis (AD) were monitored for two nights in a sleep lab. The patients wore a GENEActiv accelerometer on each wrist and were recorded with the FLIR a35 thermal camera for the duration of their sleep period. On the second in-lab night they also underwent polysomnography (PSG). Scratching and restless movements recorded by the thermal camera were labeled by trained annotators.
Research sample	45 AD patients were recruited as part of a larger research effort (determined by the criteria of Hanifin and Rajka, the Investigator's Static Global Assessment (ISGA; ≥2) and body surface area (BSA; ≥ 5%) obtained at screening (0 to 30 days prior to visit 1); aged 31.7 +/- 16.01 years [12-63; range]; sex: 16 (35.5%) male). Participants were also required to have active pruritus as determined by PROs: Peak Pruritus Numerical Rating Scale (ppNRS21 (Instrument copyrighted by Regeneron and Sanofi-Aventis); ≥3) and Severity of Pruritus Scale (SPS; ≥1) at screening, and permitted to continue concomitant AD treatments during the study.
Sampling strategy	A sample size of 40 subjects was chosen to enable us to estimate the mean percent error in scratching duration between video and actigraphy assessments with a 95% confidence interval margin of error of 5%, assuming a standard deviation of 17.4. This standard deviation is based on previous data from Moreau, et al, 2017; we expect a similar standard deviation in this cohort. Due to technical issues with data collection for several subjects near study start, an additional 5 subjects were added via an amendment.
Data collection	Overnight video recordings were performed with the FLIR a35 thermal camera to allow for maximal detection of all restless or scratching movement (including under covers). Videos were labeled for scratching and restless behaviors by two trained annotators, and an arbitrator if there were differences in annotation. Accelerometry was collected with GENEActiv accelerometers from both wrists of each subject from the beginning of the first in clinic visit to the end of the second in clinic visit continuously. Polysomnography was collected on the second in clinic visit and was performed by trained study staff.
Timing	8/6/2018 - 3/21/2019
Data exclusions	12 participants excluded due to missing data required for algorithm development and evaluation. (Missing video recordings. Missing device data. Issues with time alignment of device data and video recordings.) Time alignment issues resulted in a mismatch between labeled scratch and restless events and the corresponding wearable device data, which prevented us from generating ground truth labels for some subject nights and/or entire subjects.
Non-participation	50 subjects were screened for the study, while 45 subjects completed the study.
Randomization	Subjects were not allocated into experimental groups.

Reporting for specific materials, systems and methods

We require information from authors about some types of materials, experimental systems and methods used in many studies. Here, indicate whether each material, system or method listed is relevant to your study. If you are not sure if a list item applies to your research, read the appropriate section before selecting a response.

Materials & experimental systems

n/a	Involved in the study	
☒	☐	Antibodies
☒	☐	Eukaryotic cell lines
☒	☐	Palaeontology and archaeology
☒	☐	Animals and other organisms
☐	☒	Human research participants
☒	☐	Clinical data
☒	☐	Dual use research of concern

Methods

n/a	Involved in the study	
☒	☐	ChIP-seq
☒	☐	Flow cytometry
☒	☐	MRI-based neuroimaging
