# Supplementary material for: Development of digital measures for nighttime scratch and sleep using wrist-worn wearable devices
Source: NPJ Digit Med. 2021 Mar 3;4:42. doi: 10.1038/s41746-021-00402-x (PMC7930047; doi:10.1038/s41746-021-00402-x)
Supplement: Supplementary file 2 — Supplementary Information [file 41746_2021_402_MOESM2_ESM.pdf]

Supplementary Material for “Development of digital measures for nighttime scratch and sleep using wrist-worn wearable devices”

Supplementary Table 1. Further details on participant exclusions for this analysis. V01, V02 refer to in lab overnight visit 1 and visit 2 respectively.

**Failure Mode**	**Number of occurrences (per wrist)**	**Night affected (PSG exclusive to V02)**
No accelerometer data (device configuration error)	5 (2 subjects excluded; 1 subject provided only 1 wrist of data)	V01, V02
Watches removed	2 (1 subject excluded)	V02
Excessive accelerometer clock drift	4 (2 subjects excluded)	V01, V02
No reference videos	2 (1 subject excluded)	V01, V02
No annotations during sleep window (subject not in frame, lens cap not removed)	4 (2 subjects excluded)	V01, V02
Unable to align sensor data (no alignment event, weak/undetectable alignment event, alignment event performed off camera)	10 (5 subjects excluded)	V01, V02

Supplementary Table 2. Scratch classifier leave-one-subject-out validation performance per subject.

**Subject**	**Accuracy**	**Sensitivity**	**Specificity**	**Positive Predictive Value**	**Negative Predictive Value**	**F1 Score**
**001**	0.825	0.556	0.878	0.469	0.910	0.508
**002**	0.811	0.833	0.784	0.826	0.793	0.829
**003**	0.750	0.619	0.807	0.587	0.827	0.603
**004**	0.852	0.907	0.717	0.889	0.754	0.898
**005**	0.596	0.466	0.811	0.804	0.477	0.590
**006**	0.777	0.457	0.948	0.825	0.766	0.588
**007**	0.766	0.701	0.843	0.840	0.706	0.764
**008**	0.810	0.548	0.881	0.553	0.879	0.550
**009**	0.596	0.490	0.703	0.623	0.578	0.549
**010**	0.708	0.662	0.796	0.861	0.552	0.749
**011**	0.725	0.365	0.828	0.377	0.821	0.371
**012**	0.854	0.829	1.000	1.000	0.500	0.906
**013**	0.769	0.587	0.902	0.813	0.750	0.682
**014**	0.654	0.662	0.648	0.632	0.676	0.647
**015**	0.608	0.511	0.750	0.750	0.511	0.608
**016**	0.734	0.745	0.706	0.863	0.526	0.800
**017**	0.803	0.705	0.882	0.827	0.788	0.761
**018**	0.675	0.339	0.901	0.698	0.670	0.457
**019**	0.820	0.702	0.911	0.859	0.799	0.772

**020**	0.655	0.500	0.677	0.184	0.903	0.269
**021**	0.695	0.537	0.811	0.674	0.706	0.598
**022**	0.704	0.782	0.642	0.633	0.789	0.699
**023**	0.744	0.661	0.840	0.827	0.682	0.734
**024**	0.578	0.444	0.765	0.724	0.497	0.550
**025**	0.782	0.479	0.929	0.767	0.786	0.590
**026**	0.786	0.883	0.667	0.764	0.824	0.819
**027**	0.813	0.660	0.882	0.717	0.851	0.688
**028**	0.545	0.537	0.556	0.611	0.479	0.571
**029**	0.515	0.362	0.752	0.693	0.433	0.476
**030**	0.700	0.700	0.689	0.976	0.112	0.816
**031**	0.793	0.699	0.865	0.800	0.788	0.746
**032**	0.722	0.664	0.789	0.787	0.667	0.720
**033**	0.773	0.701	0.829	0.762	0.780	0.730
**Total**	0.734	0.669	0.815	0.822	0.659	0.738

Note: Total performance metrics computed by combining all participant epochs together.

Supplementary Table 3. Scratch classifier leave-one-subject-out validation performance (median and interquartile range) by disease severity at screening (ISGA; obtained up to 30 days prior to study start).

	Mild	Moderate	Severe
n	9	20	4
Accuracy	0.73 [0.70, 0.77]	0.74 [0.67, 0.79]	0.83 [0.76, 0.85]
Sensitivity	0.54 [0.46, 0.59]	0.66 [0.53, 0.70]	0.83 [0.74, 0.85]
Specificity	0.83 [0.80, 0.88]	0.80 [0.69, 0.88]	0.80 [0.77, 0.86]
PPV	0.69 [0.62, 0.82]	0.76 [0.63, 0.81]	0.86 [0.82, 0.92]
NPV	0.71 [0.58, 0.77]	0.78 [0.63, 0.80]	0.63 [0.49, 0.76]
F1	0.59 [0.55, 0.68]	0.69 [0.59, 0.75]	0.86 [0.77, 0.90]

Note: median and interquartile ranges were calculated using R 4.0.2 and the package "*tableone" 0.12.0*

Supplementary Table 4. Scratch classifier leave-one-subject-out validation performance (median and interquartile range) by sex.

	FEMALE	MALE
n	23	10
Accuracy	0.73 [0.69, 0.78]	0.77 [0.67, 0.81]
Sensitivity	0.66 [0.48, 0.70]	0.64 [0.54, 0.69]
Specificity	0.81 [0.73, 0.87]	0.80 [0.70, 0.88]
PPV	0.80 [0.69, 0.83]	0.71 [0.59, 0.78]
NPV	0.71 [0.57, 0.79]	0.81 [0.54, 0.85]
F1	0.68 [0.59, 0.76]	0.65 [0.52, 0.75]

Note: median and interquartile ranges were calculated using R 4.0.2 and the package "*tableone" 0.12.0*

Supplementary Table 5. Features extracted for training the scratch classifier.

Feature	Description	Processed Signal Used for Computation
Root mean square (RMS) value*	The RMS value is a measure of signal energy and is correlated with amount and intensity of motion.	SVM
Signal range	Signal range provides a measure of the extremes of motion observed in a given time window of sensor data. Higher range would indicate occurrence of a large excursion in sensor values.	SVM
Signal entropy*	Signal entropy is calculated by estimating Shannon entropy of the probability mass function of a signal. Signal entropy values close to zero indicate that the signal is periodic and smooth, whereas large negative values indicate that the signal is irregular and non-periodic.	SVM, PC1, PC2
Interquartile range (IQR) of auto-covariance*	IQR of auto-covariance is a measure of long-range dependency or periodicity of a signal. Range of auto-covariance captures if the signal is periodic or irregular.	SVM, PC1, PC2
Skewness*	Skewness is a measure of asymmetry in a signal.	SVM, PC1, PC2

Dominant frequency value*	Dominant frequency value is the value of the frequency with the highest magnitude in the normalized power spectrum of the accelerometer signal. This feature captures the fundamental frequency of the underlying movement producing the acceleration signal.	PC1, PC2
Dominant frequency magnitude*	Dominant frequency magnitude captures the percentage of total signal energy in the dominant frequency.	PC1, PC2
Dominant frequency ratio	This feature captures periodicity of a signal by calculating the ratio of the energy in the dominant frequency component to the sum of energy in the entire frequency spectrum of a signal.	PC1, PC2
Mean cross rate*	Mean cross rate calculates the number of times the signal changes from positive to negative, normalized by total signal length.	SVM, PC1, PC2
Jerk ratio*	Calculation of smoothness of motion	SVM, PC1, PC2
Log dimensionless jerk*		SVM, PC1, PC2

Spectral Arc Length (SPARC)*		SVM, PC1, PC2
Permutation entropy*	Permutation entropy is a measure of complexity of a signal.	SVM, PC1, PC2
Spectral flatness*	Spectral flatness captures the amount of modulation or the level of consistency and ranges from 0 to 1.	PC1, PC2
Spectral entropy*	Spectral entropy is calculated by estimating Shannon entropy of the probability mass function of the power spectrum of a signal. Values closer to 1 indicate presence of white noise. Values closer to 0 indicate presence of periodicity in the signal	PC1, PC2

Note: An asterisk (*) indicates that the feature survived after the feature selection step. SVM = signal vector magnitude; PC1 = first principal component; PC2 = second principal component.

Supplementary Note 1. Details about the scratch and restless movement video annotation criteria.

General Definitions

Scratch behavior – Any repetitive rubbing or scratching of the body – including through fabric – performed with the hand or any part of the upper limb (from Ebata et al., [2001])

- Initiation Frame: First contact of any part of the hand – including fingers, nails, palm, and/or dorsum – with the scratch area, continuous with the scratching behavior.
- Termination Frame: Last contact of any part of the hand – including fingers, nails, palm, and/or dorsum – with the scratch area, or the last visible movement of the hand if there is a ≥ 3 second pause in scratching with the hand still in contact with the scratching area.

Scratch behaviors resulting from non-hand scratching – Any repetitive rubbing or scratching of the body – including through fabric – performed with *any* part of the body other than the upper limb (1) [[Length may be discussed.]]

- Initiation Frame: First contact of the scratching body part with the scratch area, continuous with the scratching behavior.
- Termination Frame: Last contact of the scratching body part with the scratch area, or it's last visible movement if there is a ≥ 3 second pause in scratching while it is still in contact with the scratching area.

Restless behaviors classified as non-scratch behavior – Any repetitive, generalized movements of the body without repetitive motion that would demonstrate scratching activity or that cannot be directly seen due to obstruction (e.g. by a blanket/sheet, by the Subject's body, etc.).

- Initiation Frame: First visible movement of the repetitive generalized movement.
- Termination Frame: Last visible movement of the repetitive generalized movement

Variations

Hand Scratch and Scratch behaviors resulting from non-hand scratching will have several variants meant to capture expected variations in the behavior (e.g. "Hand Scratch, Right Upper Extremity, Mild Intensity"). Each variation will use the same Initiation/Termination Frame criteria as the general annotation, but will require the rater to recognize characteristics of the behavior to select the correct variant. The *Scratch Area* is the region of skin being directly scratched, and includes adjacent skin of the scratch area. The *Scratching Behavior* refers to the movement of the scratching body part over the scratch area.

Both Hand Scratch and Other Scratch annotations will have the following variations:

- Scratching Area Location
  - Head, Face, and Neck (HFN)
  - Right Upper Extremity (RUE)
  - Left Upper Extremity (LUE)
  - Torso – including Back (TB)
  - Right Lower Extremity (RLE)
  - Left Lower Extremity (LLE)

Scratch will have the following variations:

- Scratching Intensity
  - Mild – below elbow movement only (1)
  - Moderate – includes elbow movement (2)
  - Severe – whole limb movement (3)

Annotation Template Rationale

Annotation definitions were generated from studies that applied video annotation to sleep-time scratching in atopic dermatitis. Specifically, definitions and categorizations of scratch behavior were derived from previous research[1-3]. Noro et al.'s delimiters were used to define the initiation and termination frames [4]. As specific definition and annotation methods were vaguely reported in the literature, additional details needed for consistent annotation were interpolated based on the conceptual framework established by these articles.

Supplementary Figure 1. Agreement of sleep endpoints predicted from left and right wrist.

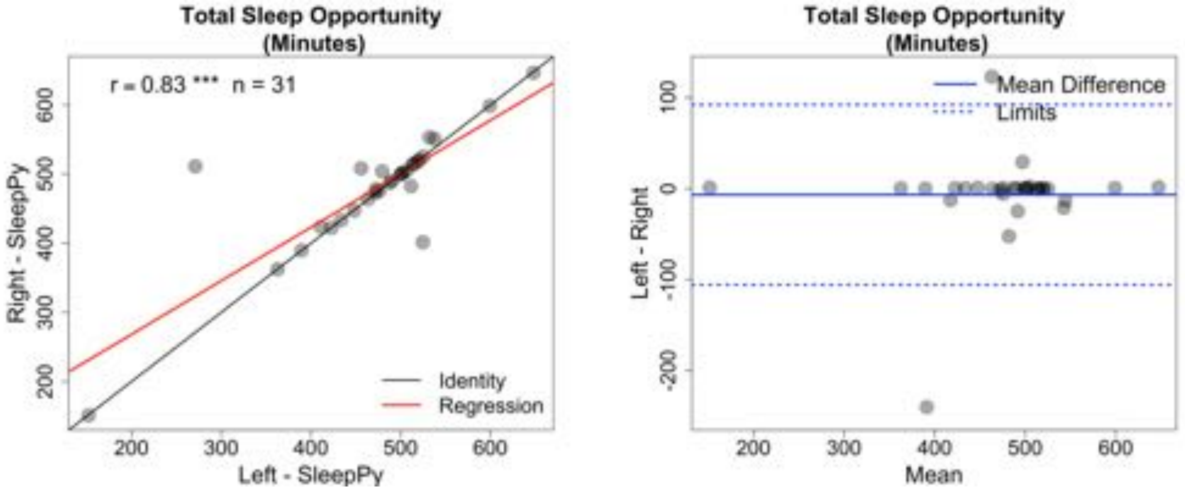

1a. Agreements of TSO between left and right wrists. The left panel shows the scatter plot and correlations along with statistical significance (significance code: 0 ‘***’ 0.001 ‘**’ 0.01 ‘*’ 0.05 ‘.’ 0.1 ‘ ’ 1). The right panel shows the Bland-Altman plot. The blue solid line represents the mean difference (bias), and the blue dotted lines represent the upper and lower limits.

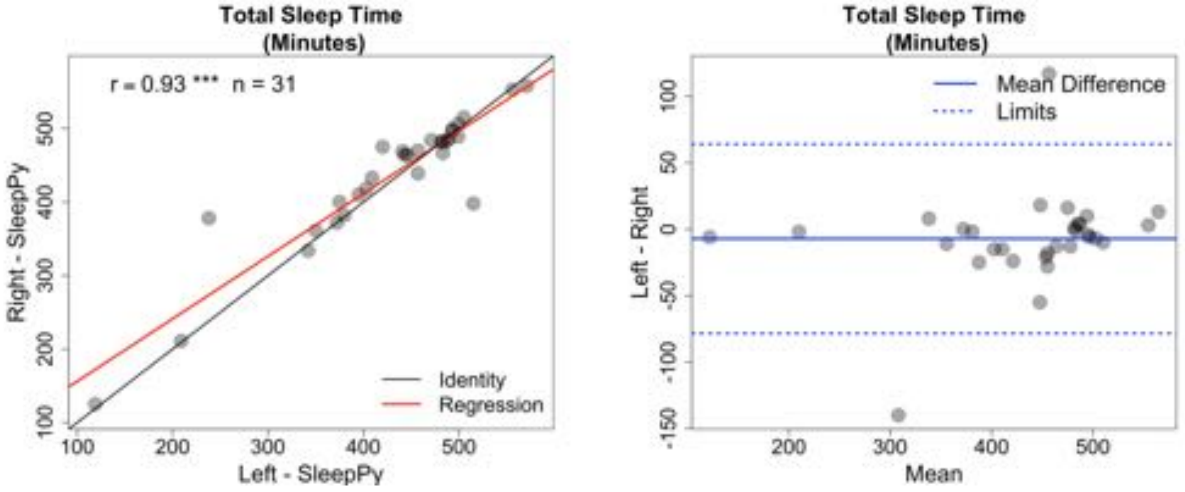

1b. Agreements of TST between left and right wrists. The left panel shows the scatter plot and correlations along with statistical significance (significance code: 0 ‘***’ 0.001 ‘**’ 0.01 ‘*’ 0.05 ‘.’ 0.1 ‘ ’ 1). The right panel shows the Bland-Altman plot. The blue solid line represents the mean difference (bias), and the blue dotted lines represent the upper and lower limits.

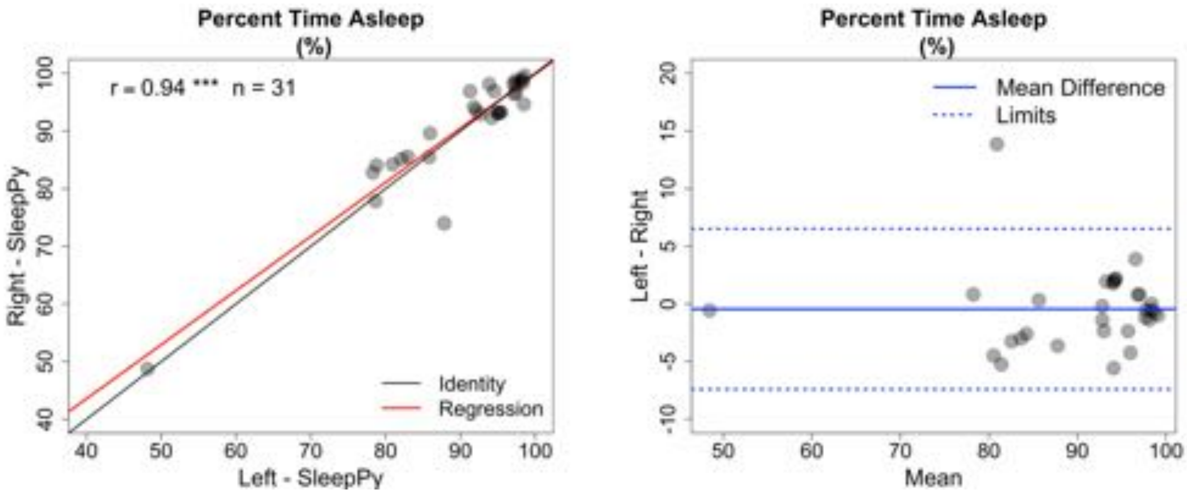

1c. Agreements of PTA between left and right wrists. The left panel shows the scatter plot and correlations along with statistical significance (significance code: 0 ‘***’ 0.001 ‘**’ 0.01 ‘*’ 0.05 ‘.’ 0.1 ‘ ’ 1). The right panel shows the Bland-Altman plot. The blue solid line represents the mean difference (bias), and the blue dotted lines represent the upper and lower limits.

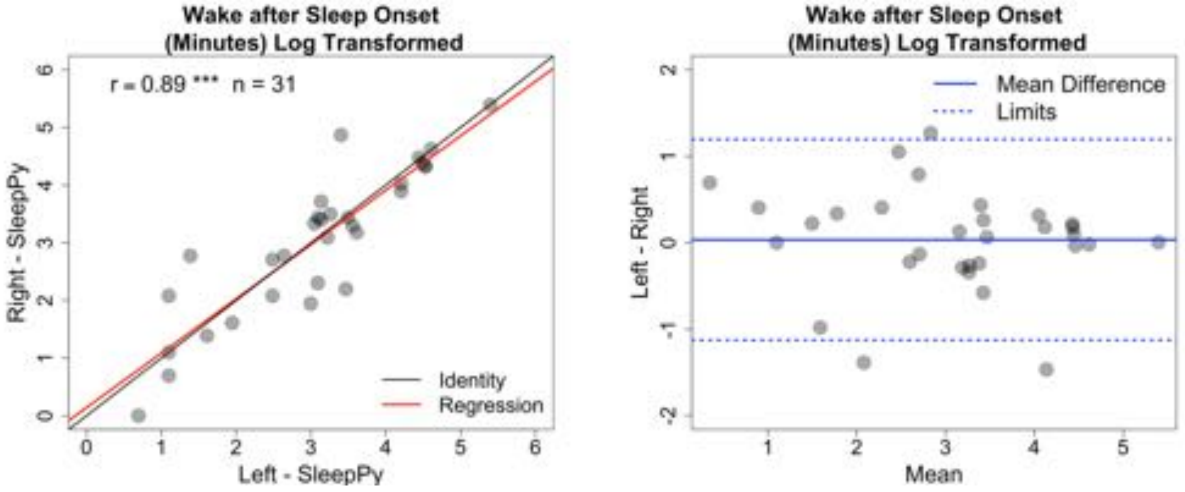

1d. Agreements of wake after sleep onset between left and right wrists. The left panel shows the scatter plot and correlations along with statistical significance (significance code: 0 ‘***’ 0.001 ‘**’ 0.01 ‘*’ 0.05 ‘.’ 0.1 ‘ ’ 1). The right panel shows the Bland-Altman plot. The blue solid line represents the mean difference (bias), and the blue dotted lines represent the upper and lower limits.

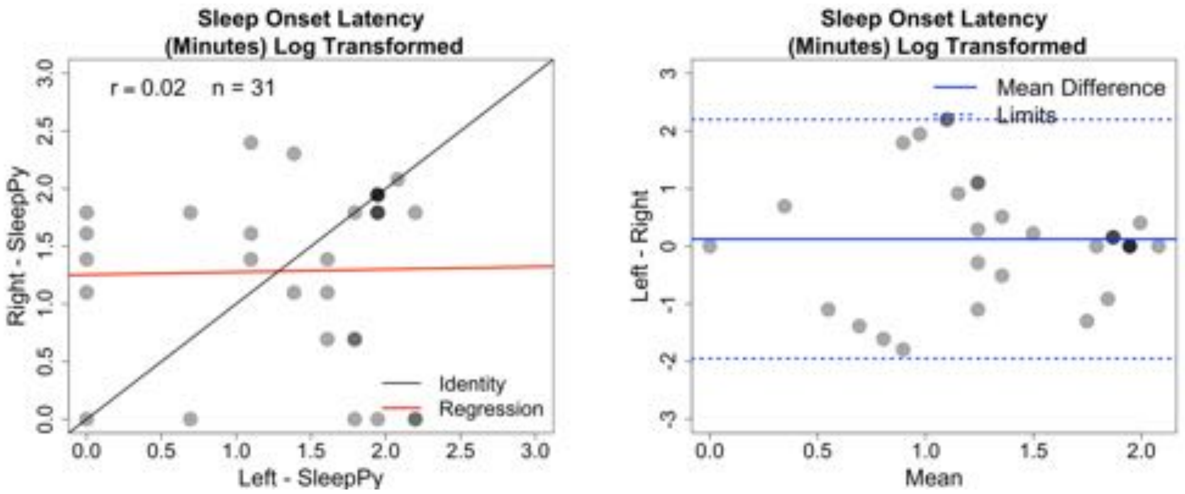

1e. Agreements of sleep onset latency between left and right wrists. The left panel shows the scatter plot and correlations along with statistical significance (significance code: 0 ‘***’ 0.001 ‘**’ 0.01 ‘*’ 0.05 ‘.’ 0.1 ‘ ’ 1). The right panel shows the Bland-Altman plot. The blue solid line represents the mean difference (bias), and the blue dotted lines represent the upper and lower limits.

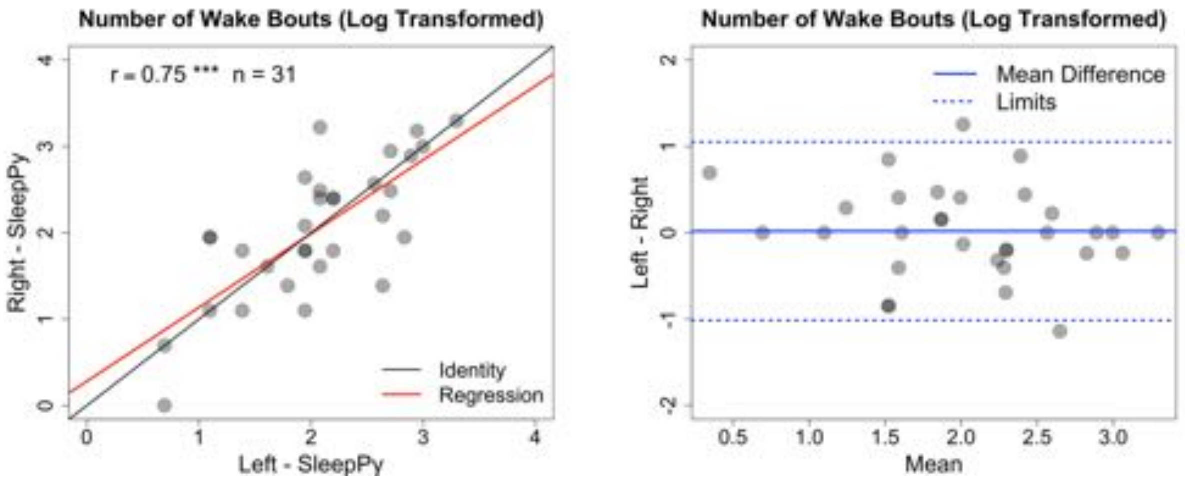

1f. Agreements of number of wake bouts between left and right wrists. The left panel shows the scatter plot and correlations along with statistical significance (significance code: 0 ‘***’ 0.001 ‘**’ 0.01 ‘*’ 0.05 ‘.’ 0.1 ‘ ’ 1). The right panel shows the Bland-Altman plot. The blue solid line represents the mean difference (bias), and the blue dotted lines represent the upper and lower limits.

Supplementary Figure 2. Aggregated predicted sleep endpoints (average of left and right) vs. PSG.

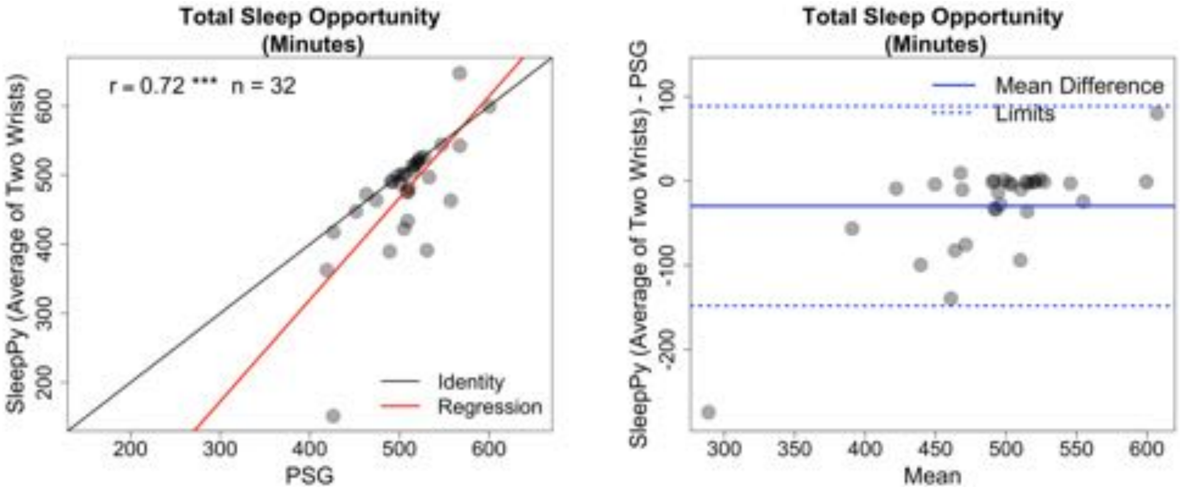

2a. Validation of TSO from sleep module prediction against PSG. The left panel shows the scatter plot and correlations along with statistical significance (significance code: 0 ‘***’ 0.001 ‘**’ 0.01 ‘*’ 0.05 ‘.’ 0.1 ‘ ’ 1). The right panel shows the Bland-Altman plot. The blue solid line represents the mean difference (bias), and the blue dotted lines represent the upper and lower limits.

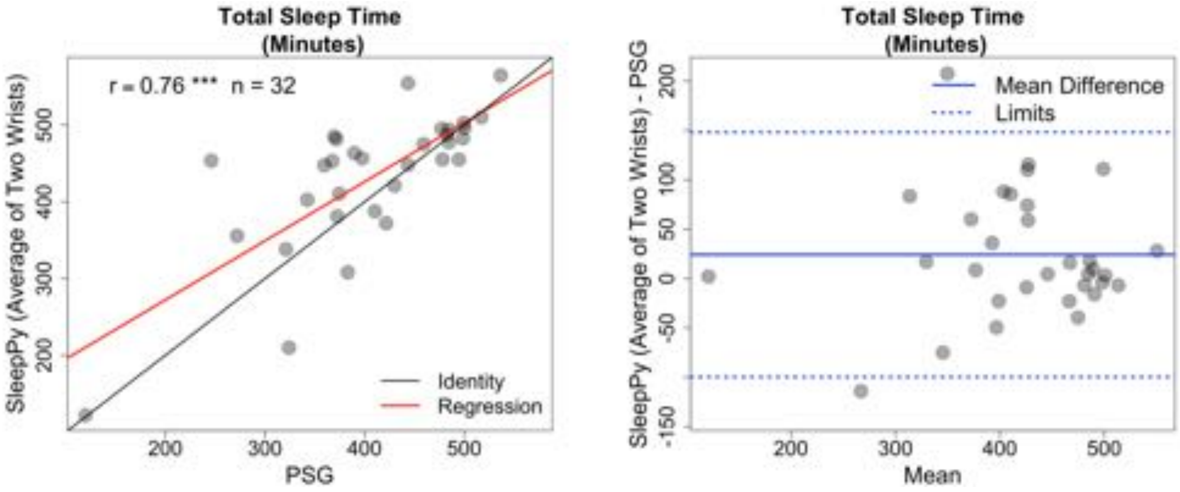

2b. Validation of TST from the sleep module prediction against PSG. The left panel shows the scatter plot and correlations along with statistical significance (significance code: 0 ‘***’ 0.001 ‘**’ 0.01 ‘*’ 0.05 ‘.’ 0.1 ‘ ’ 1). The right panel shows the Bland-Altman plot. The blue solid line represents the mean difference (bias), and the blue dotted lines represent the upper and lower limits.

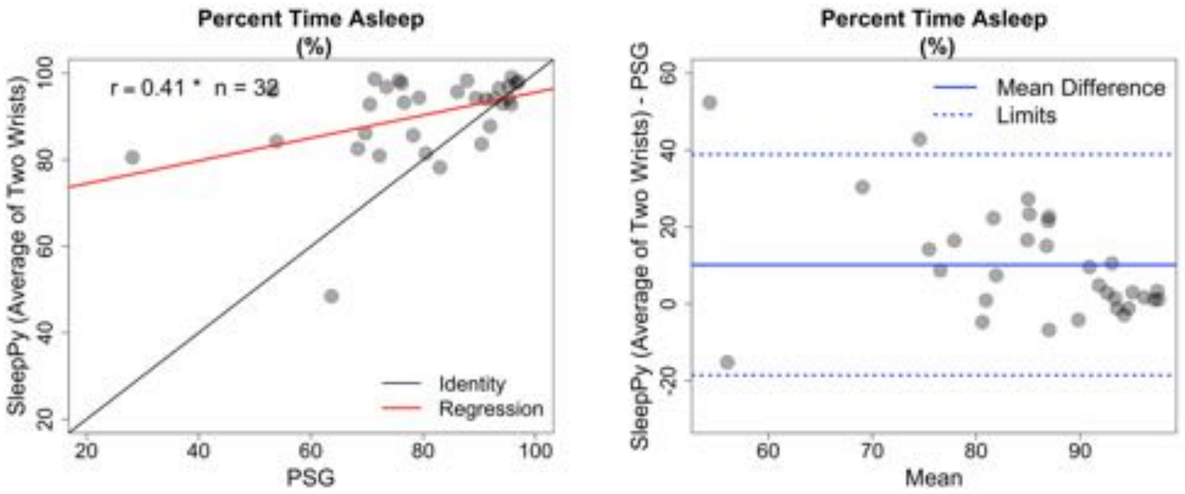

2c. Validation of PTA from the sleep module prediction against PSG. The left panel shows the scatter plot and correlations along with statistical significance (significance code: 0 '***' 0.001 '**' 0.01 '*' 0.05 '.' 0.1 ' ' 1). The right panel shows the Bland-Altman plot. The blue solid line represents the mean difference (bias), and the blue dotted lines represent the upper and lower limits.

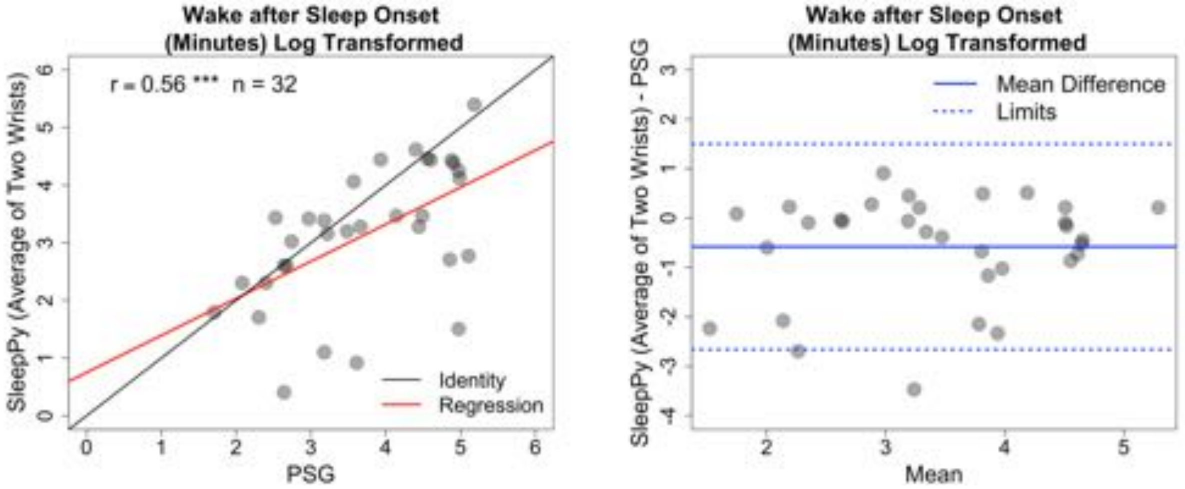

2d. Validation of wake after sleep onset from the sleep module prediction against PSG. The left panel shows the scatter plot and correlations along with statistical significance (significance code: 0 '***' 0.001 '**' 0.01 '*' 0.05 '.' 0.1 ' ' 1). The right panel shows the Bland-Altman plot. The blue solid line represents the mean difference (bias), and the blue dotted lines represent the upper and lower limits.

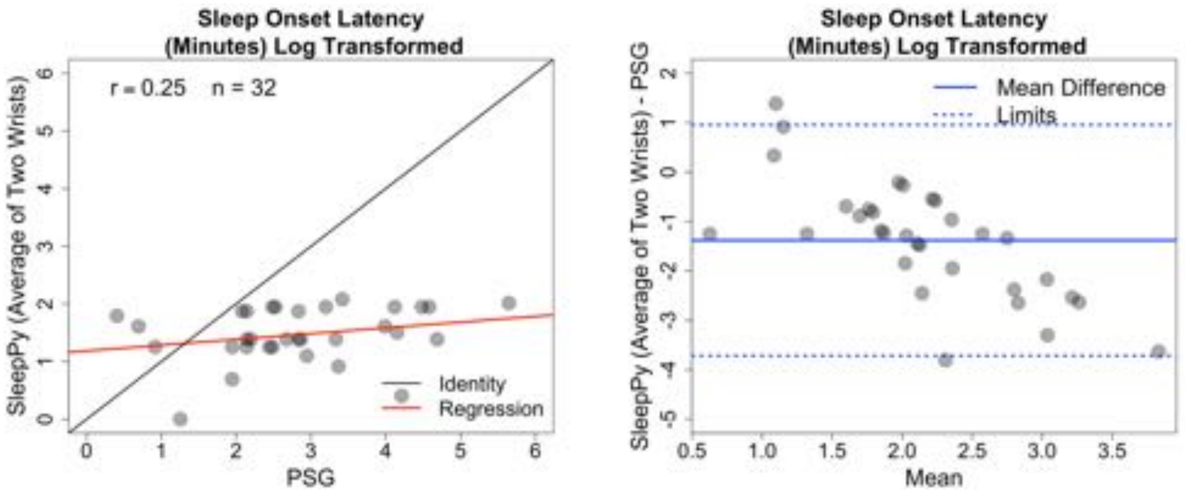

2e. Validation of sleep onset latency from the sleep module prediction against PSG. The left panel shows the scatter plot and correlations along with statistical significance (significance code: 0 ‘***’ 0.001 ‘**’ 0.01 ‘*’ 0.05 ‘.’ 0.1 ‘ ’ 1). The right panel shows the Bland-Altman plot. The blue solid line represents the mean difference (bias), and the blue dotted lines represent the upper and lower limits.

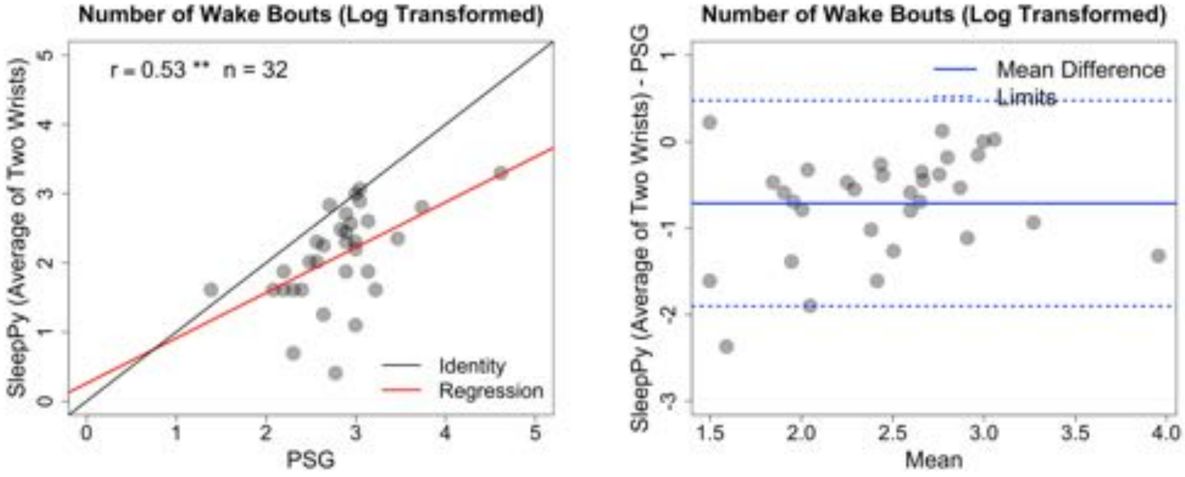

2f. Validation of wake after sleep onset from the sleep module prediction against PSG. The left panel shows the scatter plot and correlations along with statistical significance (significance code: 0 ‘***’ 0.001 ‘**’ 0.01 ‘*’ 0.05 ‘.’ 0.1 ‘ ’ 1). The right panel shows the Bland-Altman plot. The blue solid line represents the mean difference (bias), and the blue dotted lines represent the upper and lower limits.

Supplementary Figure 3. Compare predicted sleep endpoints (from left and right wrist separately) to PSG.

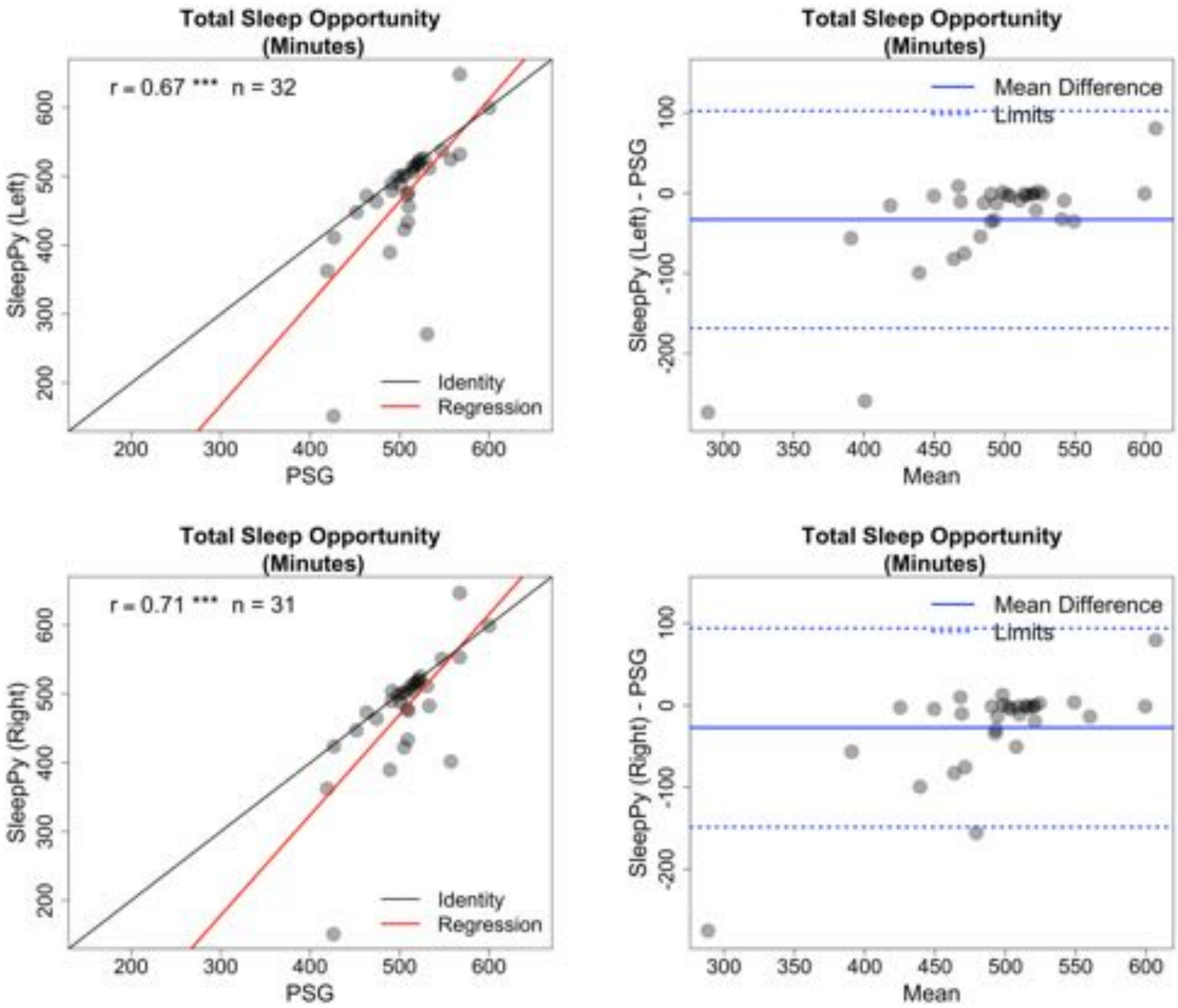

3a. Validation of TSO from the sleep module prediction (Row 1: sleep prediction from left wrist, Row 2: sleep prediction from right wrist) against PSG. The left panel shows the scatter plot and correlations along with statistical significance (significance code: 0 '***' 0.001 '**' 0.01 '*' 0.05 '.' 0.1 ' ' 1). The right panel shows the Bland-Altman plot. The blue solid line represents the mean difference (bias), and the blue dotted lines represent the upper and lower limits.

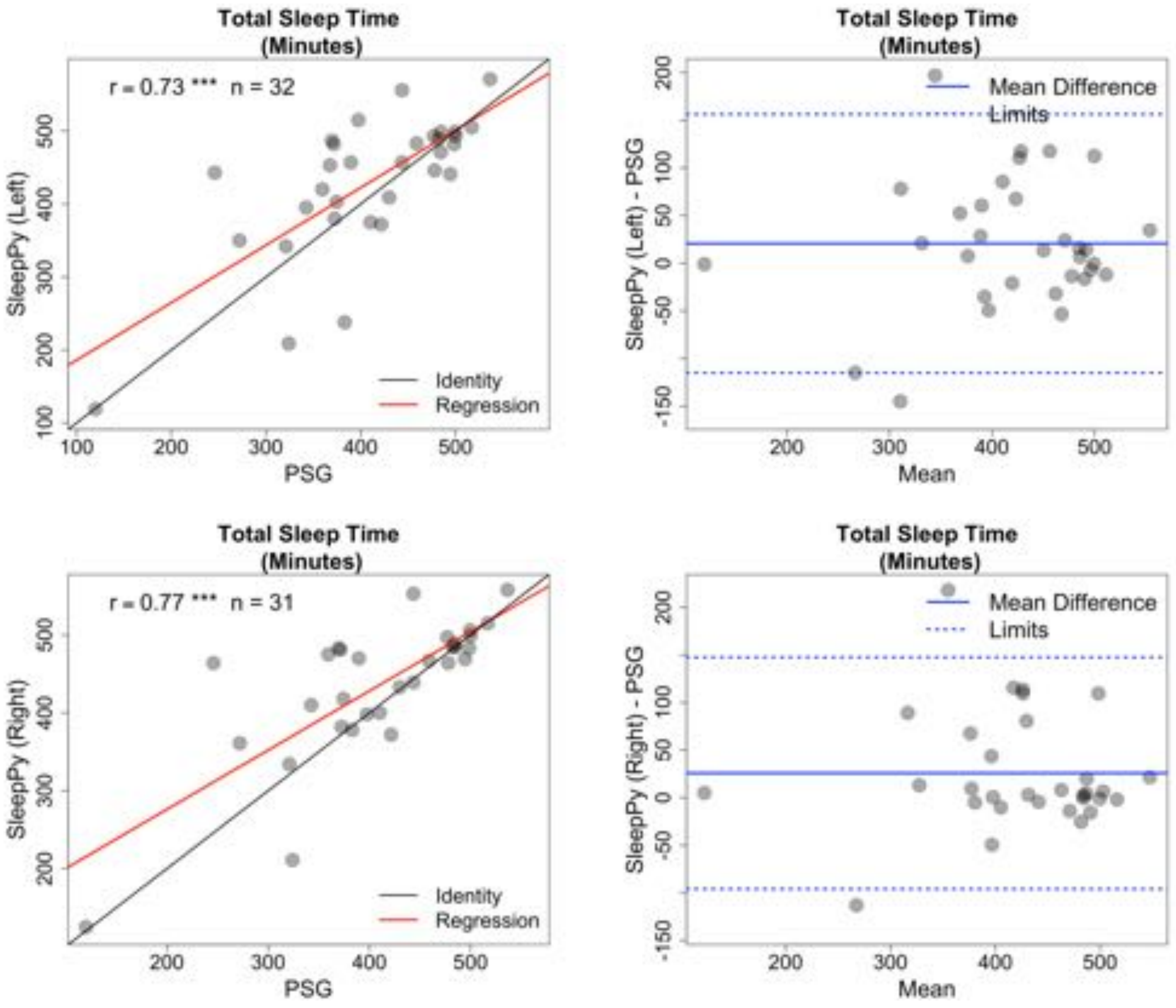

3b. Validation of TST from the sleep module prediction (Row 1: sleep prediction from left wrist, Row 2: sleep prediction from right wrist) against PSG. The left panel shows the scatter plot and correlations along with statistical significance (significance code: 0 ‘***’ 0.001 ‘**’ 0.01 ‘*’ 0.05 ‘.’ 0.1 ‘ ’ 1). The right panel shows the Bland-Altman plot. The blue solid line represents the mean difference (bias), and the blue dotted lines represent the upper and lower limits.

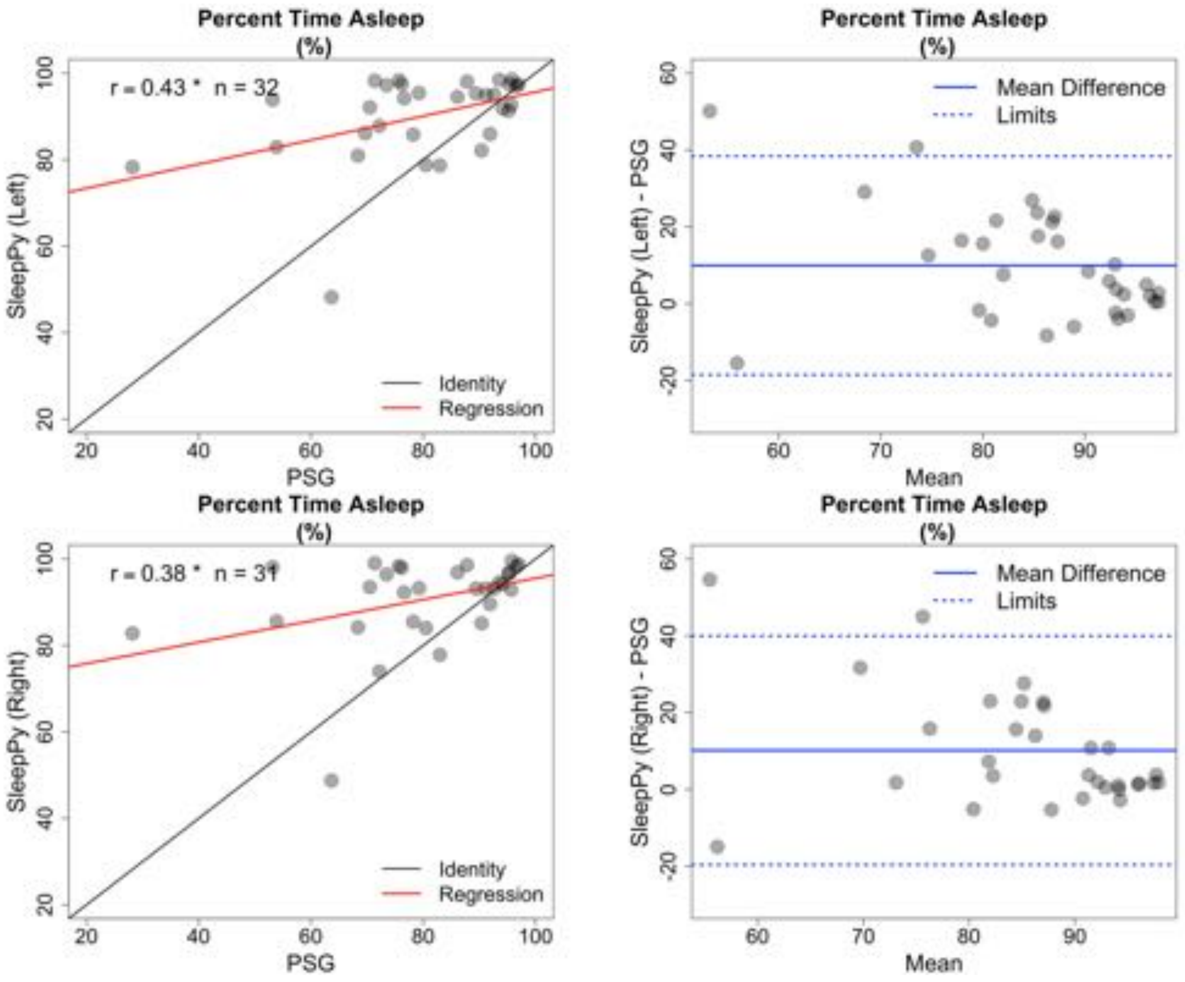

3c. Validation of PTA from the sleep module prediction (Row 1: sleep prediction from left wrist, Row 2: sleep prediction from right wrist) against PSG. The left panel shows the scatter plot and correlations along with statistical significance (significance code: 0 '***' 0.001 '**' 0.01 '*' 0.05 '.' 0.1 ' ' 1). The right panel shows the Bland-Altman plot. The blue solid line represents the mean difference (bias), and the blue dotted lines represent the upper and lower limits.

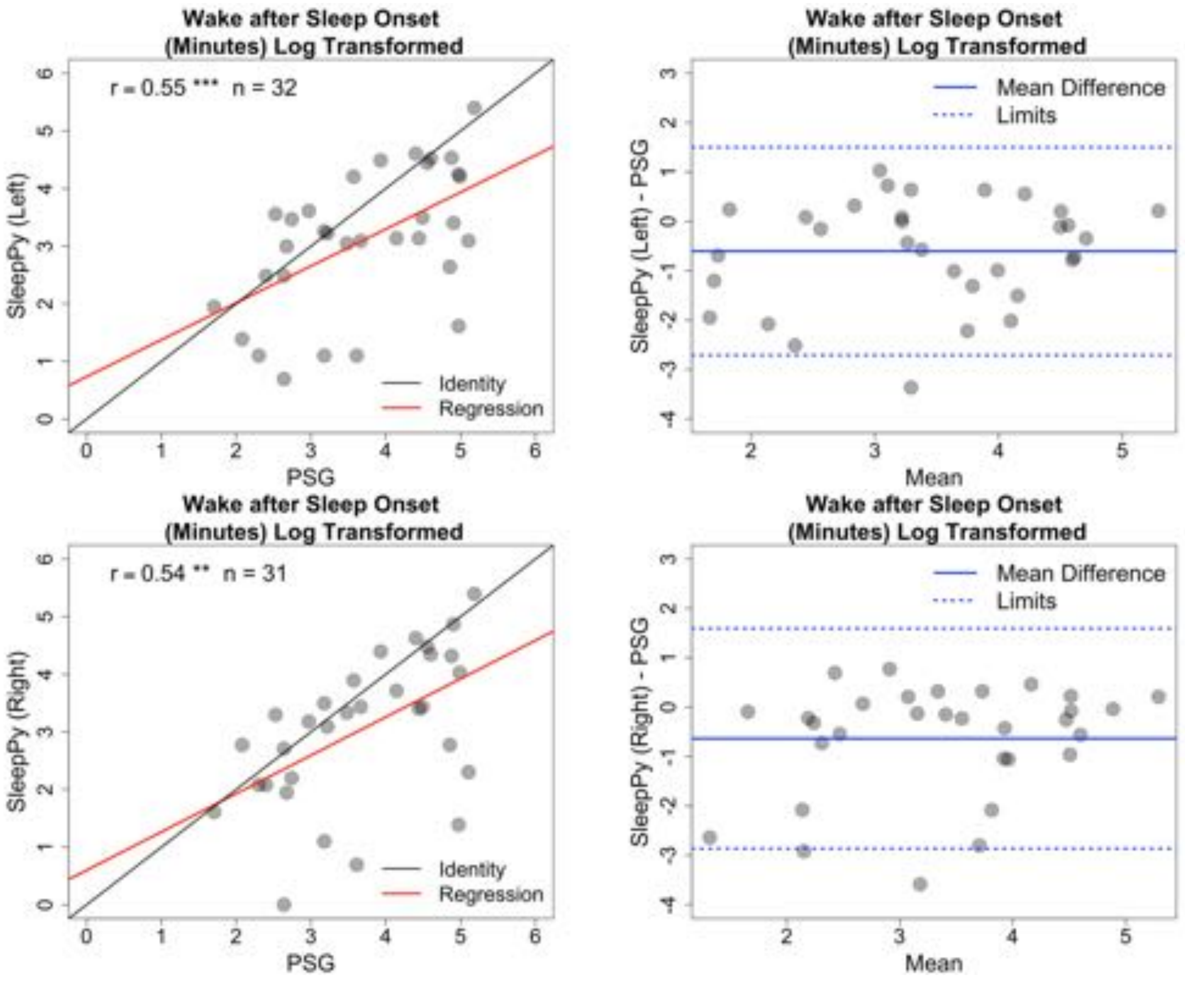

3d. Validation of wake after sleep onset from the sleep module prediction (Row 1: sleep prediction from left wrist, Row 2: sleep prediction from right wrist)) against PSG. The left panel shows the scatter plot and correlations along with statistical significance (significance code: 0 '***' 0.001 '**' 0.01 '*' 0.05 '.' 0.1 ' ' 1). The right panel shows the Bland-Altman plot. The blue solid line represents the mean difference (bias), and the blue dotted lines represent the upper and lower limits.

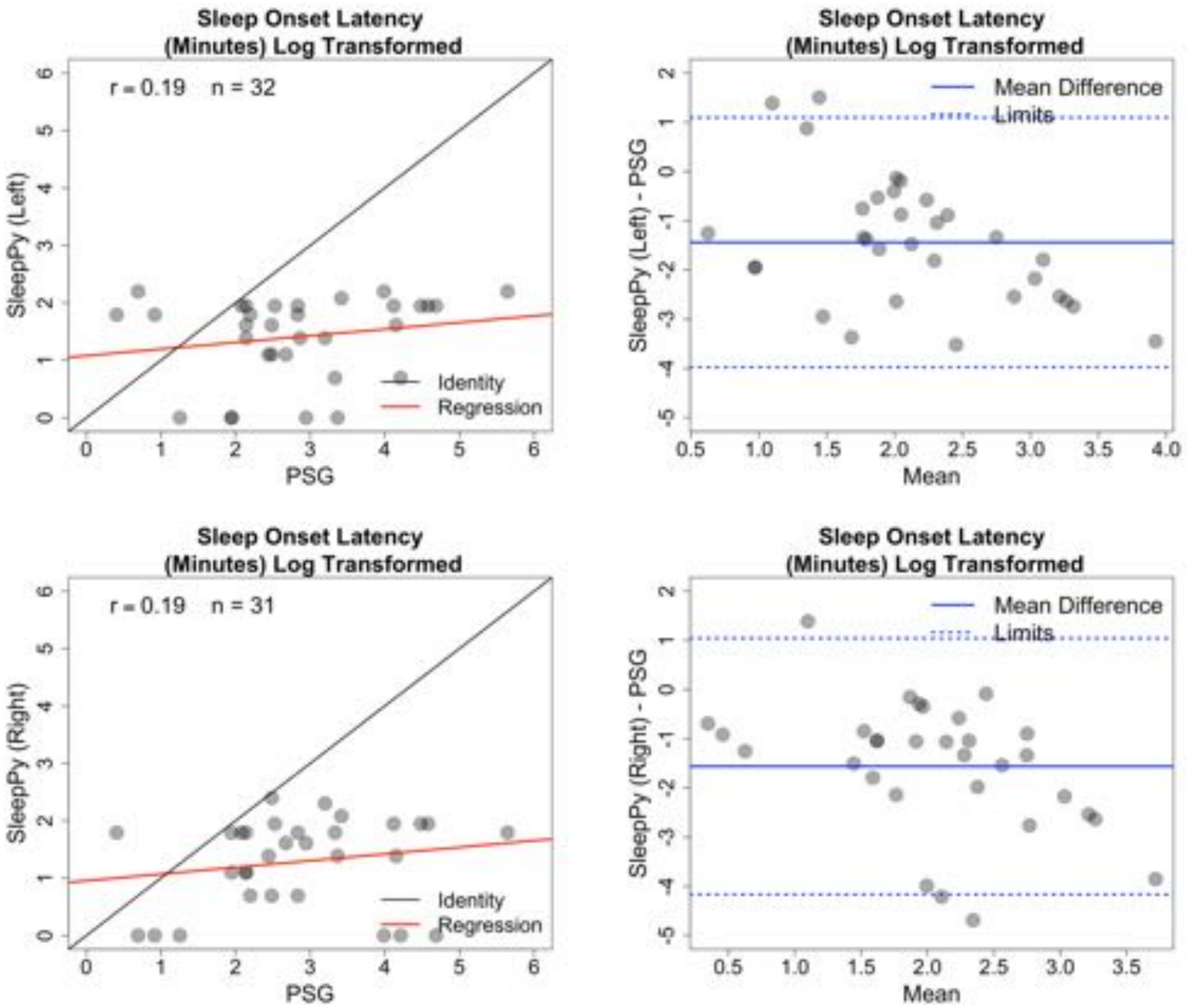

3e. Validation of sleep onset latency from wake after sleep onset prediction (Row 1: sleep prediction from left wrist, Row 2: sleep prediction from right wrist)) against PSG. The left panel shows the scatter plot and correlations along with statistical significance (significance code: 0 ‘***’ 0.001 ‘**’ 0.01 ‘*’ 0.05 ‘.’ 0.1 ‘ ’ 1). The right panel shows the Bland-Altman plot. The blue solid line represents the mean difference (bias), and the blue dotted lines represent the upper and lower limits.

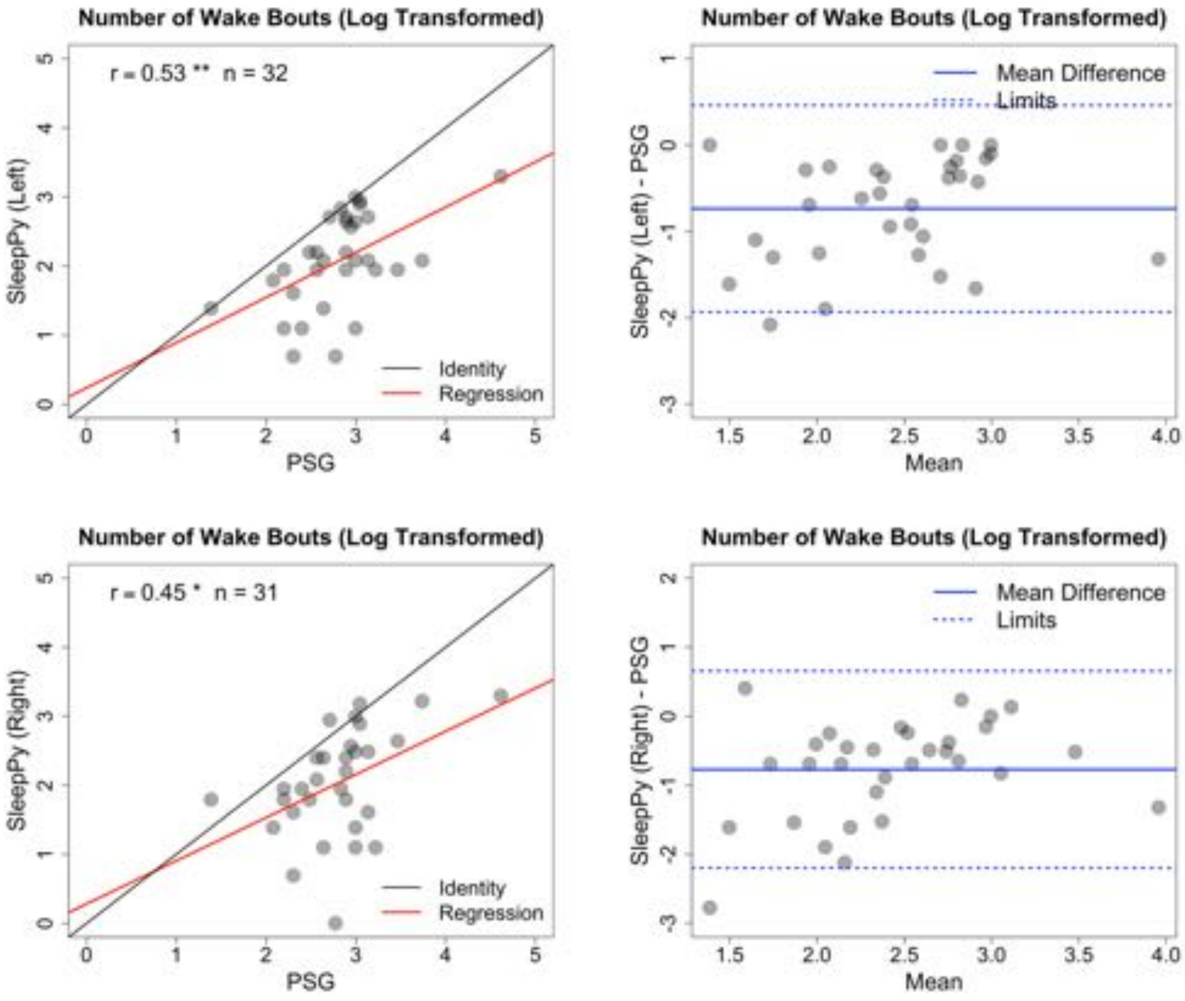

3f. Validation of number of wake bouts from wake after sleep onset prediction (Row 1: sleep prediction from left wrist, Row 2: sleep prediction from right wrist)) against PSG. The left panel shows the scatter plot and correlations along with statistical significance (significance code: 0 '***' 0.001 '**' 0.01 '*' 0.05 '.' 0.1 ' ' 1). The right panel shows the Bland-Altman plot. The blue solid line represents the mean difference (bias), and the blue dotted lines represent the upper and lower limits.

Supplementary Figure 4. Compare predicted scratch events/duration to video annotation for both first and second nights (video annotation segmented using the sleep module predicted TSO).

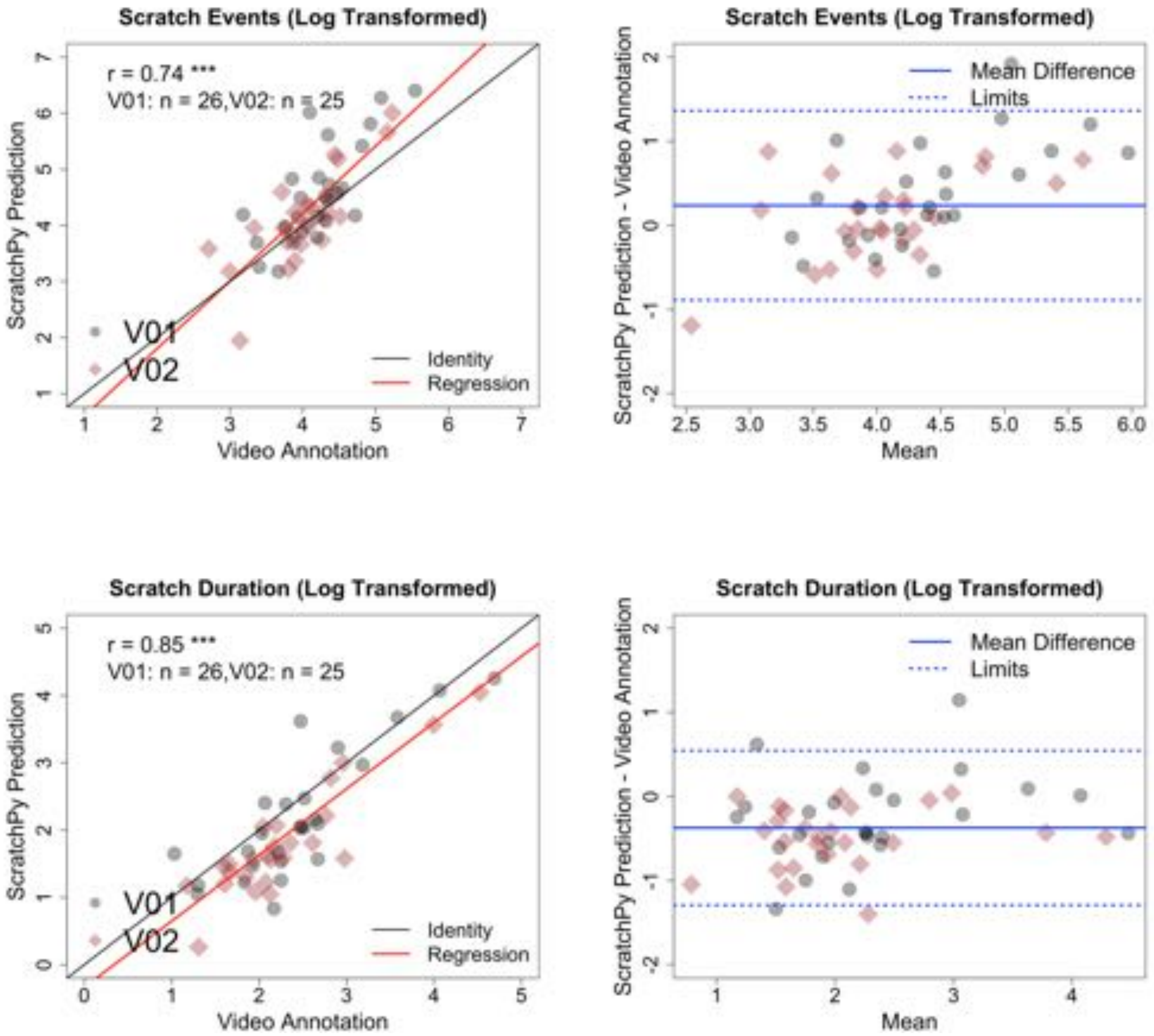

Validation of scratch counts and duration (log transformed due to skewedly distributed data) from scratch module prediction against video annotation (both first and second nights, r = 0.74 p < 0.001, r = 0.85 p < 0.001, respectively). V01 and V02 refer to first and second night, respectively. Mixed effects models were used to incorporate repeated measures[5]. Video annotation data were segmented using the sleep module predicted TSO window. Data from the first two visits were combined and shown together. The left panel shows the scatter plot and correlations along with statistical significance (significance code: 0 '***' 0.001 '**' 0.01 '*' 0.05 '.' 0.1 ' ' 1). The right panel shows the Bland-Altman plot. The blue solid line represents the mean difference (bias), and the blue dotted lines represent the upper and lower limits. These analyses validate the scratch endpoints only as video annotation data were segmented using sleep module predicted TSO instead of PSG defined TSO.

Supplementary Figure 5. Correlations between scratch endpoints and sleep endpoints.

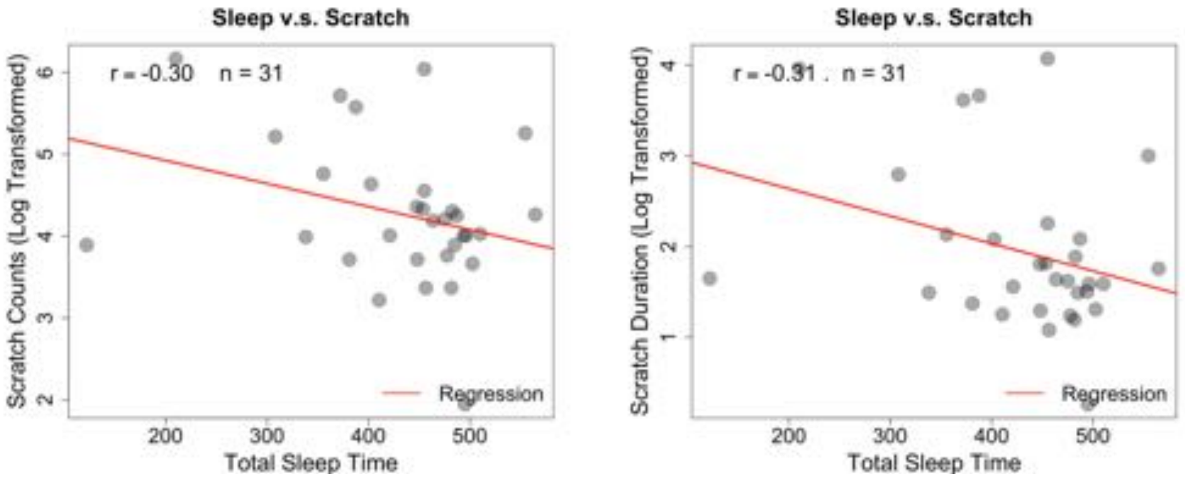

5a. Correlation between scratch endpoints (counts and durations) and TST (for sleep quantity).

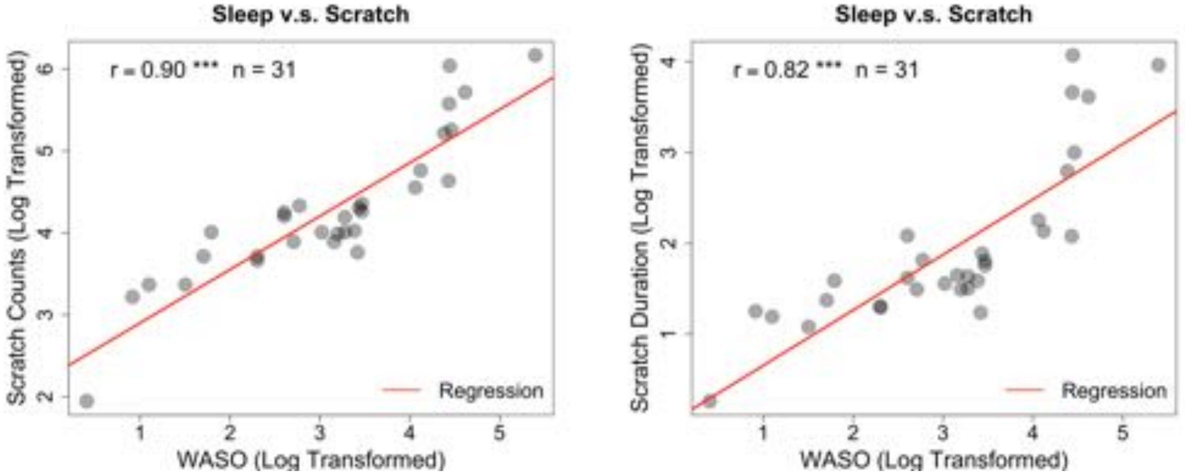

5b. Correlation between scratch endpoints (counts and durations) and WASO (sleep quality).

Supplementary Figure 6. Epoch level PSG sleep stages (sleep/wake) data for participant 8 whose predictions of TSO were not accurate.

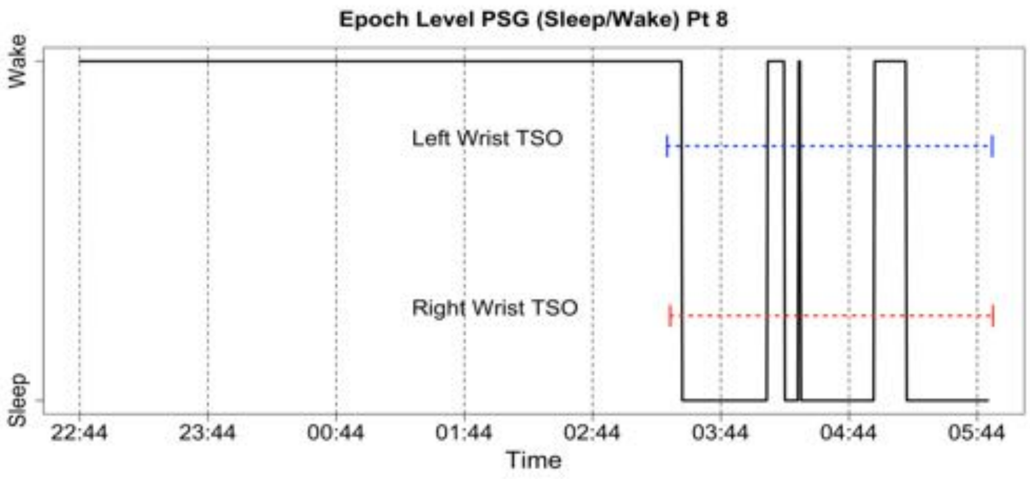

Epoch level PSG sleep stages (sleep/wake) data for participant 8. The horizontal dotted blue and red lines indicate the TSO detected by the sleep module for both wrists. Among all the sleep endpoints, TSO played a key role. Firstly, it determines the key window where sleep-wake predictions are meaningful and all other sleep endpoints are extracted. Secondly, it determines the window where scratch behavior will be predicted. Therefore, we furthered explored patient 8 whose predictions were significantly off as compared to PSG. The TSO from PSG is defined based on light off/light on time. Apparently patient 8 stayed awake for an extensive amount of time after lights-off. This explained the deviation between prediction and truth. What is promising is that the predicted TSO was close to the actual sleep interval for this patient.

Supplementary Figure 7. Distribution of temperature and movement-based threshold from empirical data.

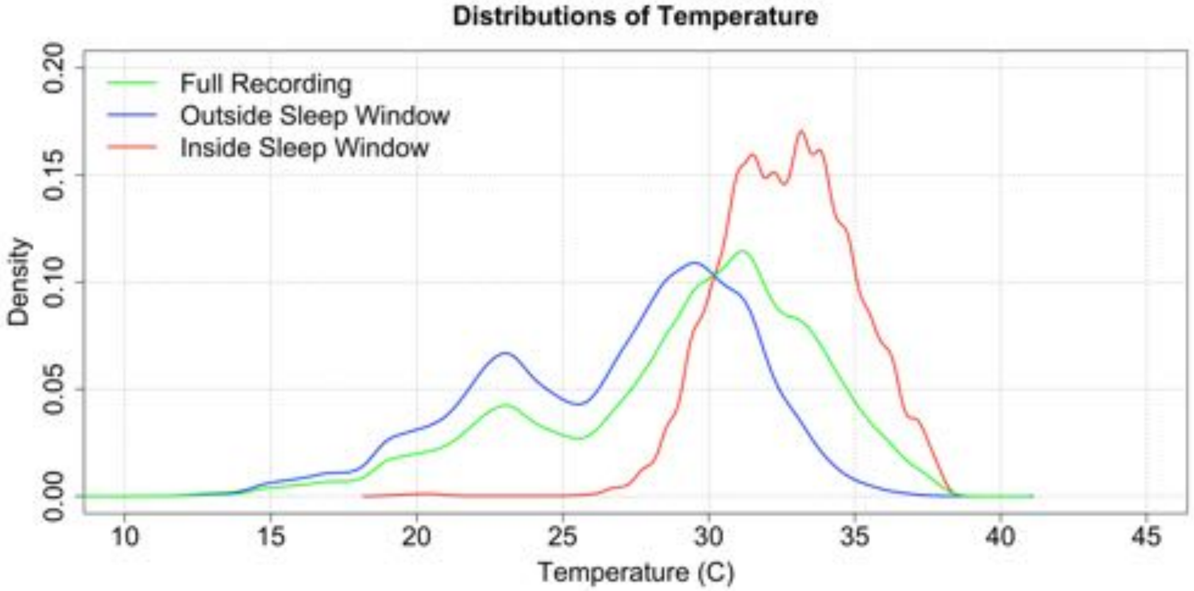

7a. Probability densities estimated in different windows (inside PSG determined sleep window, outside PSG determined sleep window, and full recording of V02). Data from all subjects with accelerometry records (N = 42) are combined. Temperature is higher within the sleep window. The threshold 25°C for temperature is chosen based on distribution.

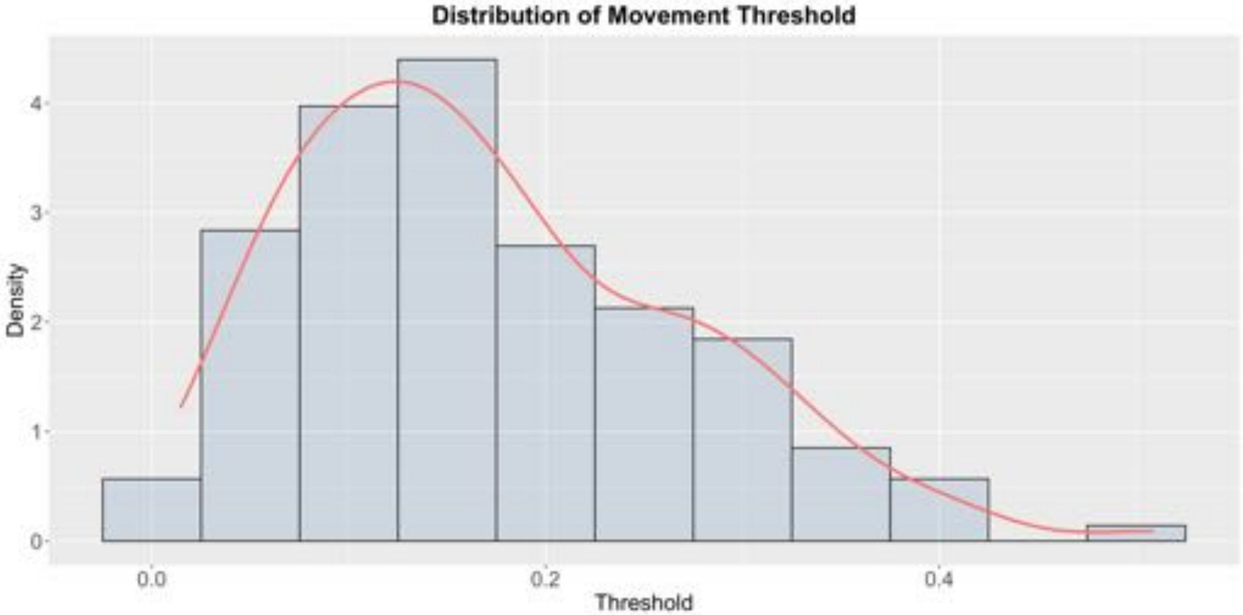

7b. Probability density for motion-based arm-angle threshold. Arm-angle threshold (z-angle threshold) is calculated as a scaled version of the 10th percentile value of all Δ z-angle values in a 24-hour period. When off-body periods are included in this calculation, the resulting threshold is artificially low, which can cause only non-wear periods to be detected as rest, or, in the more extreme case, can cause no rest periods to be detected at all. The above distribution was used

to derive a minimum viable z-angle threshold by examining the distribution of all valid thresholds across subjects with no off-body periods. The bottom 25th percentile value (approximately 0.1) was chosen to account for the scenario when off-body periods make the calculated threshold unusable, but also for the scenario when a subject has an atypically inactive recording. A threshold set at the absolute minimum of the working threshold distribution would often be too low for a typical activity level paired with significant off-body periods, and the 25th percentile value was empirically found to be a good compromise.

Supplementary References

1. Ebata T, Iwasaki S, Kamide R, Niimura M. Use of a wrist activity monitor for the measurement of nocturnal scratching in patients with atopic dermatitis. Br J Dermatol [Internet]. 2001 Feb;144(2):305–9. Available from: http://doi.wiley.com/10.1046/j.1365-2133.2001.04019.x
2. Ebata, Aizawa, Kamide, Niimura. The characteristics of nocturnal scratching in adults with atopic dermatitis. Br J Dermatol [Internet]. 1999 Jul;141(1):82–6. Available from: http://doi.wiley.com/10.1046/j.1365-2133.1999.02924.x
3. Benjamin K, Waterston K, Russell M, Schofield O, Diffey B, Rees JL. The development of an objective method for measuring scratch in children with atopic dermatitis suitable for clinical use. J Am Acad Dermatol [Internet]. 2004 Jan;50(1):33–40. Available from: https://linkinghub.elsevier.com/retrieve/pii/S0190962203024800
4. Noro Y, Omoto Y, Umeda K, Tanaka F, Shiratsuka Y, Yamada T, et al. Novel acoustic evaluation system for scratching behavior in itching dermatitis: Rapid and accurate analysis for nocturnal scratching of atopic dermatitis patients. J Dermatol [Internet]. 2014 Mar;41(3):233–8. Available from: http://doi.wiley.com/10.1111/1346-8138.12405
5. Nakagawa, S., Schielzeth, H. A general and simple method for obtaining R^2 from Generalized Linear Mixed-effects Models. Methods in Ecology and Evolution. 2013l; 4: 133–142.
